# Modeling proximalisation in axolotl limb regeneration

**DOI:** 10.1038/s41598-025-10527-8

**Published:** 2025-07-24

**Authors:** Hernán Arce, Alberto Sebastián Ceccarelli, Rodrigo Carlos Córdoba, Ana Catarina Rodrigues Oliveira, Maximina Hee Yun, Osvaldo Chara

**Affiliations:** 1 Instituto de Tecnología, Universidad Argentina de la Empresa, Buenos Aires, Argentina; 2 School of Biosciences, University of Nottingham, Sutton Bonington Campus, Nottingham, LE12 5RD UK; 3 Chinese Institutes for Medical Research, Beijing, China; 4 CRTD Center for Regenerative Therapies, Technische Universität Dresden, Dresden, Germany; 5 Physics of Life Excellence Cluster, Dresden, Germany

**Keywords:** Biophysics, Computational biology and bioinformatics, Developmental biology, Systems biology

## Abstract

The axolotl (*Ambystoma mexicanum*) possesses a remarkable ability to regenerate tissues. Following limb amputation, a blastema of progenitor cells forms, expands, and reconstructs all distal structures, implying that mature cells near the wound retain positional memory along the proximal–distal (PD) axis. Key regulators of positional identity, such as Prod1 and Tig1, promote proximalisation—a shift toward a more proximal identity—when overexpressed, but the mechanisms governing this process remain unclear. In this study, we tracked changes in cellular density along the PD axis of regenerating axolotl limbs after transfecting distal blastemas with Tig1 and Prod1, mapping the spatiotemporal distribution of transfected cells and their progeny throughout regeneration. Using a continuous mathematical modelling approach, we predict a proximalisation velocity induced by factors eliciting proximal identity as Prod1 and Tig1, which is consistent with a proximalisation force driven by a positional potential. Our findings provide a foundational framework for understanding how cells acquire positional identity to guide limb regeneration in axolotls.

## Introduction

Tissue regeneration showcases nature’s ability to restore lost or damaged structures, which spans across a wide range of species, from invertebrates to vertebrates^1^. What makes an animal capable of regeneration and how this relates to its normal development are not clear^2^. In stark contrast to most mammals, which exhibit a rather limited regenerative capacity, urodele amphibians, such as the axolotl and newts, regenerate a wide range of complex structures, including the spinal cord, parts of the brain, heart tissues and the limbs throughout their lives^3^. The remarkable ability of the axolotl (*Ambystoma mexicanum*) limb to regenerate after amputation has turned it into a powerful system for studying regeneration, providing a unique opportunity to unveil the cellular and molecular processes involved^4^.

As in frog tadpoles and fish, amputation of the axolotl limb leads to the formation of a specialised structure at the injury site called the blastema, which constitutes a mass of proliferative, undifferentiated cells that arise from the dedifferentiation of mature cells within the adjacent stump tissue^5–8^, mainly from connective tissue dermal fibroblasts^9–12^. These cells, under the influence of the apical epidermal cap (AEC), a signalling hub that forms over the wound, and nerve-derived signals, re-enter the cell cycle and proliferate to replace only the lost tissue^13,14^.

The blastema is a highly organised structure that contains critical information necessary for accurate limb reconstruction. Classic proximal–distal polarity reversal experiments^15–17^ have shown that the blastema only regenerates structures distal to the level of the amputation, a phenomenon known as the rule of distal transformation^13,18^. For example, a wrist blastema will regenerate the hand, whereas a shoulder blastema will give rise to an entire arm^18–21^. These experiments suggest that the progenitor cells within the limb stump retain a memory of their original position along the proximal–distal (PD) axis. This ‘positional memory’ is preserved and interpreted during the blastema stages, enabling the autonomous regeneration of only the missing structures—a property known as positional identity^22^. This inherent ability of the blastema cells to recognise and follow positional cues ensures that regeneration occurs with the correct morphological patterning. Despite these insights, the mechanisms by which blastema cells maintain their positional identity and how this information is used to regenerate the precise amount of tissue are still not fully understood^23,24^.

Seminal grafting studies have supported the hypothesis that positional identity is encoded in a molecular gradient along the PD axis and is manifested as a cell surface property^20,25–27^. In 2002, PROD1, a glycosylphosphatidylinositol (GPI)-anchored surface protein present in regenerating newt limb tissues, was identified^28^. Similar to the newt, Prod1 expression in axolotls is distributed in a decreasing gradient along the PD axis and is upregulated by retinoic acid (RA) but interestingly, it lacks a GPI anchor domain^29–31^. When Prod1 is overexpressed in axolotl distal blastema cells—normally responsible for integrating distal structures like the hand—these cells undergo displacement and contribute to more proximal structures, such as the upper arm, in the final regenerate. This phenomenon is known as proximalisation^32^. Recently, another gene has been identified as a potential determinant of proximal positional identity, namely the transmembrane factor Tig1^33^. This retinoic acid-responsive gene encodes a cell surface molecule, and its expression follows a gradient along the PD axis during limb regeneration. Tig1 promotes cell differential affinity and induces proximalisation of distal blastema cells, likely by reprogramming them towards a proximal-like identity, through the regulation of key genes such as Prod1 and Meis1^33^. However, how the positional memory system in the axolotl limb blastema is affected by Prod1 and Tig1 after amputation, leading to proximalisation, remains to be elucidated.

This study quantitatively investigates proximalisation induced by factors eliciting proximal identity, like Prod1 or Tig1, during axolotl limb regeneration. We performed image analysis of distal blastema cells overexpressing Tig1 or Prod1 at sequential time points during regeneration, and extracted their spatio-temporal distribution along the PD axis within regenerating limbs using the newly developed Meandros software. To analyse these distributions, we developed a partial differential equation (PDE) of a reaction–diffusion-advection (RDA) mathematical model that introduces a proximalisation velocity as a readout of the proximalisation dynamics resulting from changing positional cues due to Tig1 or Prod1 overexpression. Fitting the model to the experimental data allowed us to infer this velocity. We hypothesise that the proximalisation velocity is caused by a proximalisation force, which in turn is derived from a proximalisation potential. Our work provides a theoretical framework for analysing the underlying basis of proximalisation, central to achieving faithful regeneration.

## Results

### Tig1 and Prod1 overexpression promotes cell-autonomous proximal displacement in the regenerating limb

To investigate the dynamics of the proximalisation mechanism in the context of a regenerating axolotl limb, we revisited previous experiments in which two proximalising factors, Prod1 and Tig1, were overexpressed^32,33^. In the current study, we build on the data from Oliveira et al*.* (2022) and extend our analysis to examine the temporal evolution of the position of the transformed cells along the PD axis throughout regeneration.

**Fig. 1:**
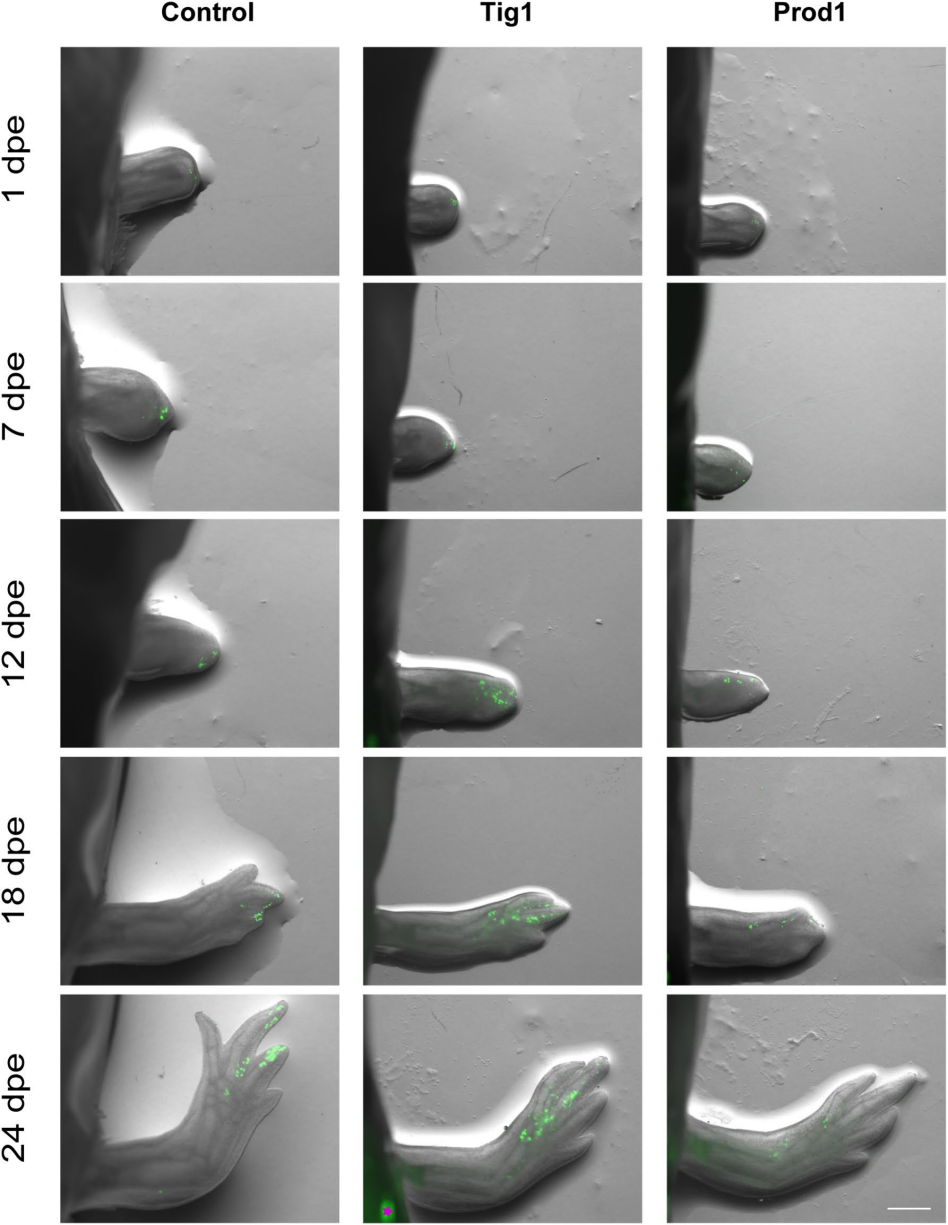
Tig1 and Prod1 overexpression elicits cell-autonomous displacement toward proximal regions during axolotl limb regeneration. Time series representing the displacement assay using control, Tig1 and Prod1 overexpression experimental conditions (columns). Images were taken at 1, 7, 12, 18 and 24 days post-electroporation (dpe) (rows). Despite their initial localization in the distal-most blastema compartment, control-electroporated cells remain at the distal region of the regenerating limb, while both Tig1 and Prod1-transfected cells can shift their position towards more proximal locations along the regenerate. Asterisk indicates unspecific autofluorescence in the trunk. Scale bar = 1 mm.

In these experiments, the distal compartment of 4-day blastemas was electroporated with either Gfp (Control), Tig1 + Gfp or Prod1 + Gfp plasmid combinations. The labelled cells were then monitored at 1, 7, 12, 18 and 24 days post-electroporation and their distribution along the proximal–distal axis analysed (Supplementary Fig. 1). Our observations show that both Tig1 and Prod1 overexpression resulted in a notable proximal translocation of GFP^+^ cells in comparison to the control condition, in which GFP^+^ cells largely remain in distal locations (Fig. 1). Changes in relative positions are apparent as early as the 12th day time point and a marked distinction is visible between control and the Prod1 and Tig1 conditions by the 18th day time point (Fig. 1). Overexpression of Tig1 and Prod1 results in distinct phenotypes, with some of the Prod1-transformed cells achieving the longest displacement distances along the PD axis by the end of regeneration, remarkably including displacement to regions beyond the amputation plane. On the other hand, Tig1-overexpressing cells tended to overall exhibit lesser spatial dispersion than Prod1-overexpressing ones.

To gain deeper insight into the spatio-temporal dynamics of electroporated cells and their progeny during limb regeneration, we went beyond these qualitative observations by performing quantitative analysis of the experimental data through image analysis.

### Image analysis allows quantification of the proximalisation effect driven by Prod1 and Tig1

**Fig. 2:**
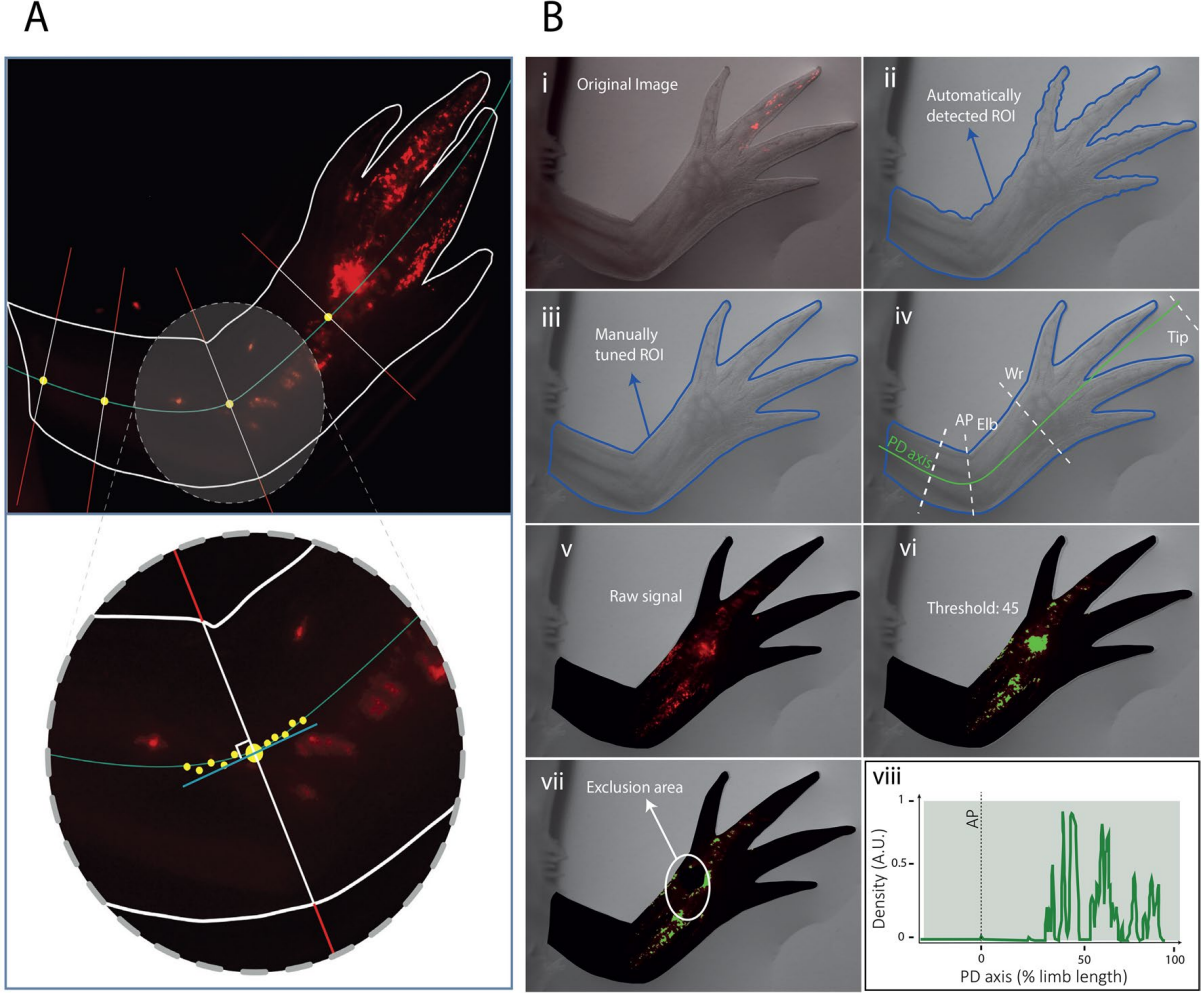
Meandro’s pipeline enables analysis of electroporated cells and their progeny in regenerating limbs. (**A**) Top: Algorithm scheme for image analysis. Bottom: Detail of the upper image. At each position along the proximal–distal (PD) axis, the local tangent is calculated based on nearby points along the axis. Subsequently, the orthogonal line is computed, and the number of pixels that have an intensity above the threshold. This information is then assigned to the corresponding position along the axis. The outcome is a one-dimensional representation of the signal in the limb. (**B**) Software pipeline: (**i–iii**) the Region Of Interest (ROI) is detected by AI and fine-tuned by the user. (**iv**) the user sets the PD axis manually. (**v–vii**) the software reads fluorescent signals above a user-defined threshold. (**viii**) cell density along the PD axis plot. *AP* Amputation plane.

In order to quantitatively investigate how overexpression of Prod1 and Tig1 affects the spatial and temporal position of electroporated cells and their progeny, we aimed to extract their spatial distribution along the PD axis from the microscopy images acquired at the mentioned time points. A first and direct observation of the morphology of the axolotl limb anticipated a major challenge to this task: the PD axis exhibits a curvature that becomes more pronounced as the regeneration process progresses (Fig. 1). Therefore, any attempt to analyse the spatio-temporal distribution of fluorescence signals along the proximal–distal dimension should be performed in relation to a curved axis. To this end, we developed Meandros, a software whose primary function is to collapse the two-dimensional space of microscopy images into a single spatial dimension, in this case the PD axis (Fig. 2A,B). The software uses AI tools to extract the region of interest (ROI) from the brightfield channel of the microscopy image. The PD axis, along which the intensity profile of the image is analysed, is set via the Graphical User Interface (GUI) (Fig. 2A,B). The intensity threshold of the fluorescence signal is set on the fluorescence channel to subtract the background signal, and artefacts can be manually excluded using the exclusion area tools (see Materials and Methods "Image Analysis to determine the cell density profile from fluorescent microscopy images" and Supplementary Information, Sect. 2.2 for more details). Briefly, to collapse the two-dimensional space of the image and assign the fluorescence signal to the single dimension of interest, the PD axis in this study, our algorithm takes the local derivative at each point of the curved axis using the adjacent spatial positions (Fig. 2A,B). The fluorescence signal within the orthogonal line is then summed and the result is assigned to the current position of the axis. This quantity can be normalised in various ways; in this study, normalisation was performed relative to the maximum intensity collected over the entire analysis space (see details in Materials and methods "Blastema generation, plasmid electroporation and imaging of the regeneration timeline" and Supplementary Information, Sect. 2.6).

**Fig. 3:**
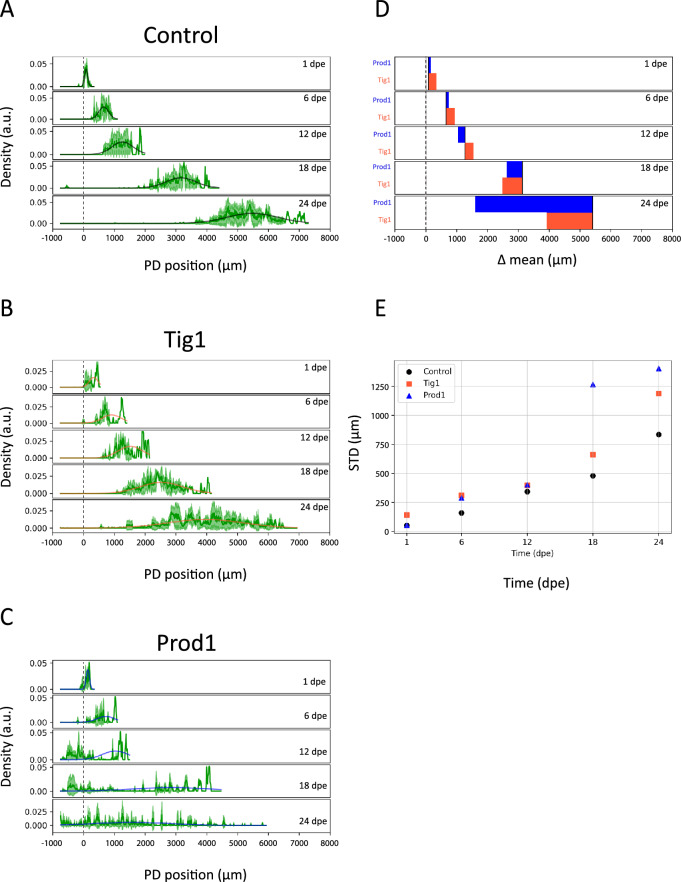
Overexpression of Tig1 and Prod1 broadens spatial cell density distributions and shifts them proximally. (**A**–**C**) Spatial distributions of labelled cells along the proximal–distal (PD) axis of the regenerating limb of the axolotl at different times post electroporation for the Control (n = 3), Tig1 (n = 4), and Prod1 (n = 4) conditions. The continuous green line and the green shaded area represent the mean and the standard deviation, respectively. The coloured continuous line represents the fit of the distributions to a Gaussian for each condition: Control (black), Tig1 (orange) and Prod1 (blue). The fits were performed by calculating the first two moments of the experimental distribution. (**D**) Difference between the means of the distributions at each time point, with the color code consistent with (**A**–**C**). (**E**) Standard deviation versus time for control (black), Tig1 (orange) and Prod1 (blue).

Using Meandros, we exported the spatial profile of the normalised density for the electroporated cells and their progeny in control experiments, as well as under Prod1 or Tig1 overexpression, at the different timepoints (Fig. 3A–C). To analyse the density profiles quantitatively and in an unbiased manner, we performed a Gaussian fit. Our results show that both Tig1 and Prod1 exhibit a shift in the Gaussian mean towards proximal locations as well as an increase in the Gaussian standard deviation compared to the control condition (Fig. 3A–E). These results quantitatively confirm what has been observed qualitatively in previous reports: overexpression of Tig1 and Prod1 causes cells to relocate to more proximal regions than those dictated by their original fate under control conditions^29,32,33^.

The area under the curve of the density profiles increases in control, Tig1 and Prod1 conditions (Supplementary Fig. 2D–F). However, the exponential rate, the inverse of which would be proportional to an average cell cycle length of the labelled cells, is not affected by Tig1 or Prod1 overexpression (Supplementary Fig. 2D–F). Consistently, the regenerated limb grows exponentially during regeneration and the corresponding exponential rate was not affected by Prod1 or Tig1 overexpression (Supplementary Fig. 2A–C), which might have been expected according to previously reported experiments^32,33^. This probably reflects the low transgene dose combined with the limited number of electroporated cells, which were not sufficient to generate widespread effects in the regenerate. In contrast, global higher doses of Tig1 result in tissue-scale defects^33^.

### Mathematical model of proximalisation in the regenerating axolotl limb

To gain a mechanistic understanding of the proximalisation effect induced by factors eliciting proximal identity as Tig1 and Prod1, we employed a modelling approach. Since cell diameters are approximately 10^–3^ the size of the limb or smaller, we adopted a continuous formalism to represent the dynamics of the density of electroporated cells and their progeny within the limb tissue during regeneration (for details see Supplementary Information, Sect. 3.1). Consistent with our Meandros-based quantification, we focused on the spatio-temporal distribution of cell density along the PD axis.

**Fig. 4:**
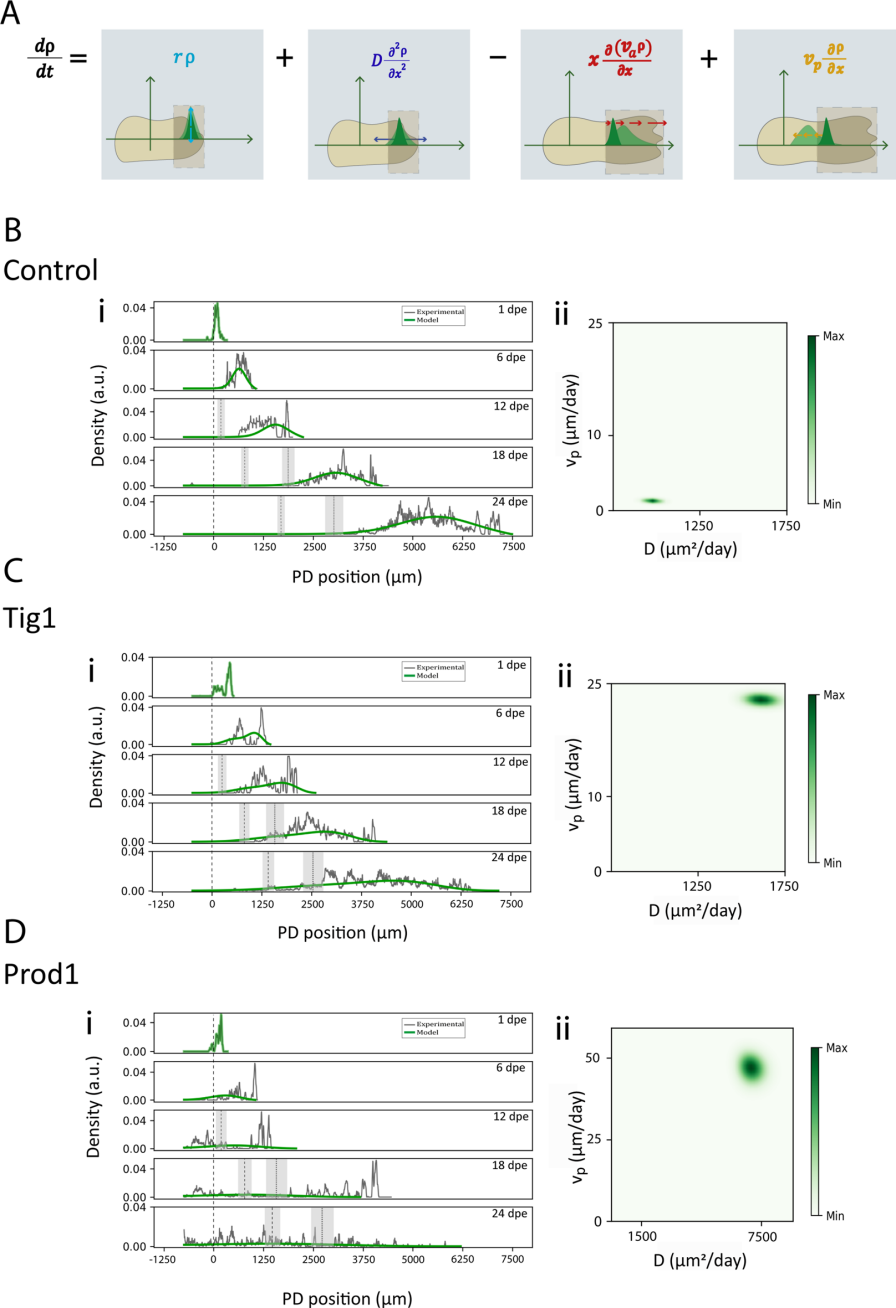
Tig1 and Prod1 overexpression increase model-predicted Proximalisation velocity and Diffusion coefficient. (**A**) Schematic representation of the Proximalisation model indicating its different terms and their effects on an ideal cellular density profile in a cartoonish limb. (**B**–**D**) (**i**) Spatial distributions of cell density for control (**B**), Tig1 (**C**) and Prod1 (**D**) along the proximal–distal (PD) axis at different days post electroporation (dpe). In grey, experimental data (Control, n = 3; Tig1, n = 4; Prod1, n = 4); in green, simulation using the Proximalisation model. The amputation plane is at the 0 μm position. The dashed vertical line indicates the mean position of the wrist, while the dotted vertical line indicates the mean position of the elbow (the shaded region represents the standard deviations). (**ii**) Posterior distributions of the parameters *v*_*p*_ and *D* for the Proximalisation model.

As our results indicate that the area under the density profiles grows exponentially (Supplementary Fig. 2D–F), we interpret this as a proliferative process and modelled it as first order kinetics, i.e., proportional to the number of cells. Furthermore, the growth of the distribution width over time, as measured by the standard deviation (Fig. 3E), suggested diffusive spreading, which we modelled accordingly. To account for the fact that the electroporated cells and their progeny move within an expanding host tissue, we introduced an advective velocity *v*_*a*_. Finally, and more importantly, we extended this idea to model the proximalisation effect induced by factors like Tig1 and Prod1 as an additional advective velocity *v*_*p*_ directed towards proximal regions, which we call the proximalisation velocity, which we notate as *v*_*p*_. All these assumptions are crystallised in the following one-dimensional reaction–diffusion–advection equation (Fig. 4A):

1 $$\frac{d\rho }{dt}=r\rho +D\frac{{\partial }^{2}\rho }{\partial {x}^{2}}-\frac{\partial \left({v}_{a}\rho \right)}{\partial x}+\frac{\partial \left({v}_{p}\rho \right)}{\partial x}$$

Which means:

2 $$\frac{d\rho }{dt}=r\rho +D\frac{{\partial }^{2}\rho }{\partial {x}^{2}}-\rho \frac{\partial {v}_{a}}{\partial x}-{v}_{a}\frac{\partial \rho }{\partial x}+\rho \frac{\partial {v}_{p}}{\partial x}+{v}_{p}\frac{\partial \rho }{\partial x}$$

where $\rho$ represents the cell density along the PD axis position $x$, $r$ is the proliferation rate, and $D$ is the diffusion coefficient. Additionally, *v*_*a*_ and *v*_*p*_ correspond to the advective and proximalisation velocities, respectively, as mentioned above.

With the following initial and boundary conditions:

3 $${\rho }\left(x,t=0\right)=f\left(x\right), \frac{\partial {\rho }}{\partial x}\left(x=0,t\right)=0, \frac{\partial {\rho }}{\partial x}\left(x=L(t),t\right)=0$$

With $f(x)$ a prescribed initial profile along the PD axis and $L(t)$ the growing tissue length.

The first reaction term on the right-hand side of Eqs. (1) and (2) encodes the average net proliferation rate of the limb tissues *r*. This parameter can be related to the average net cell cycle length of the electroporated cells (T_c_) as follows:

4 $$r=\frac{ln2}{{T}_{C}}$$

The second term on the right-hand side of Eqs. (1) and (2) corresponds to the cell diffusion process within the limb and is controlled by the cell diffusion coefficient *D*. The following term models the expansion of the host tissue with an advective velocity *v*_*a*_ as the regenerating limb grows exponentially (Supplementary Fig. 2A–C)^34^:

5 $${v}_{a}=ax$$

As a consequence, we obtain:

6 $$\frac{d\rho }{dt}=r\rho +D\frac{{\partial }^{2}\rho }{\partial {x}^{2}}-\rho a-ax\frac{\partial \rho }{\partial x}+\rho \frac{\partial {v}_{p}}{\partial x}+{v}_{p}\frac{\partial \rho }{\partial x}$$

Assuming that the proximalisation velocity along the PD axis is constant, $\frac{\partial {v}_{p}}{\partial x}=0 :$

7 $$\frac{d\rho }{dt}=\left(r-a\right)\rho +D\frac{{\partial }^{2}\rho }{\partial {x}^{2}}+\left({v}_{p}-ax\right)\frac{\partial \rho }{\partial x}$$

Thus, Eq. (7) and Eq. (3) allow us to describe the spatio-temporal distribution of electroporated cells and their progeny under the influence of proximalisation velocity during limb regeneration. Interestingly, the proximalisation velocity can be derived from a proximalisation force (see Supplementary Information, Sect. 3.2), which arises from the spatial landscape of a proximalisation potential (see Supplementary Information, Sect. 3.3) and could be associated with a chemotaxis-like process (see Supplementary Information, Sect. 3.4 and Discussion). Our hypothesis was that the proximalisation velocity would be very low in the control experiments and higher in the case of overexpression of factors as Prod1 or Tig1.

### Prod1 and Tig1 distal-to-proximal displacements can be described in terms of non-zero proximalisation velocities

To explore whether our model is sufficient to reproduce our experimental cell density spatiotemporal distributions, we undertook its parametrization. The model described by Eq. (7) has four parameters but two of them can be estimated. Specifically, we estimated the advection rate *a* and the proliferation rate *r* from the exponential rates of regenerating limb length kinetics (Supplementary Fig. 2A–C) and the time evolution of the area under the density profiles (proportional to the number of marked cells) extracted from Meandros (Supplementary Fig. 2D–F), respectively. To test our model, and also to determine the remaining parameters *v*_*p*_ and *D*, we decided to fit the model to the experimental density profiles at different times by adopting a Bayesian inference approach. With this method, we used the evidence from past time points to constrain the fitting of the future time point^35^.

Before fitting the experimental data, we performed a validation of the proposed method. For this purpose, artificial data were generated using the Reaction–Diffusion–Advection model with known values of *v*_*p*_, *D* and $\sigma$, which were then subjected to the aforementioned analysis. The method successfully retrieved the most probable values of the parameters that originated to the artificial data (Supplementary Fig. 3A,B).

When we fitted the model to the experimental spatiotemporal distribution of electroporated cells and their progeny by using the distribution of cell density at 1 dpe as the initial condition, we observed that the model captured a notable shift towards proximal positions of cells overexpressing Tig1 and Prod1, accompanied by an increase in their spreading along the PD axis when compared to the control condition (Fig. 4B–D). Our results show that Prod1 overexpression leads to an increase of both diffusion coefficient and proximalisation velocity, with both parameters being higher for Prod1 than for Tig1 (Table 1). Noteworthy, we obtained similar results (Supplementary Fig. 4A–C) when performing the fitting by using the likelihood-free method facilitated by the PyABC software, a distributed and scalable ABC-Sequential Monte Carlo (ABC-SMC) framework^36^.

**Table 1:** Summary statistics of proximalisation model parameters. The table contains the mean ± confidence interval (95%) of the parameters ***a*** and ***r***, together with the mean ± two times the standard deviation of the posterior distributions corresponding to the parameters *v*_*p*_ and *D.*

Condition	*a* (day^−1^)	*r* (day^−1^)	v_p_ (μm/day)	D (μm^2^/day)
Control	0.092 ± 0.005	0.08 ± 0.02	1.2 ± 0.2	977 ± 34
Tig1	0.087 ± 0.005	0.09 ± 0.02	22.5 ± 0.5	1617 ± 66
Prod1	0.084 ± 0.004	0.05 ± 0.03	46 ± 3	7022 ± 447

### Proximalisation velocity contributes most to model variance

After corroborating that the model is sufficient to explain the experimental spatio-temporal distribution of the density of electroporated cells and their progeny, one essential question arises: which parameters are most correlated with the obtained output? In other words, which of the input parameters contribute the most to the variability of the output^37^. To address this question, we conducted a variance-based sensitivity analysis^38^ on the four model parameters *D*, *v*_*p*_, *a* and *r*. The variance decomposition allows us to establish a ranking of which parameters contribute most to the output variance.

**Fig. 5:**
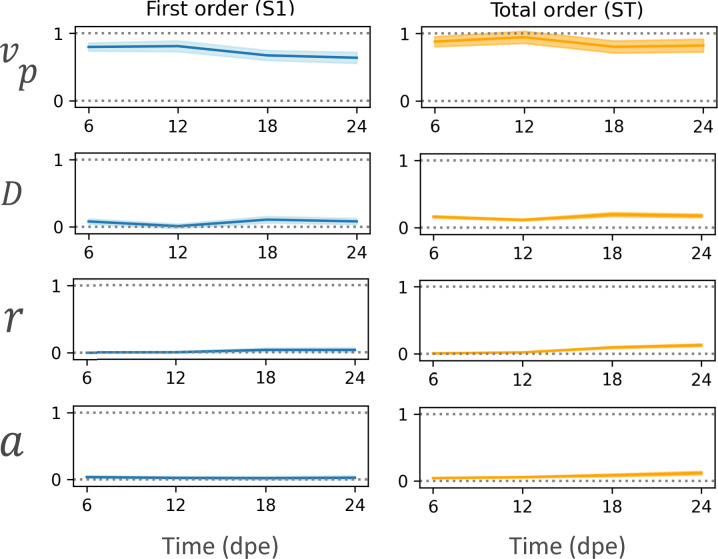
Proximalisation velocity is the primary contributor to model variance. Sobol indices for parameters $D,{v}_{p},r,  a$ as a function of time in days post electroporation (dpe). (**A**) First-order Index. (**B**) Total-order Index. Shaded areas represent the confidence interval of the indices. In all cases, the analysis was conducted evaluating the distance function defined in Materials and Methods "Posterior distributions of the parameters obtained by using pyABC" involving the experimental cell densities for the control, Tig1 and Prod1 conditions.

We calculated the first-order index that represents the contribution of each parameter to the total variance while keeping all other parameters fixed, and the global sensitivity index which corresponds to the total contribution of each parameter, including interactions between parameters. Our results indicate that the proximalisation velocity has the highest contribution to the total variance displayed in the best-fitting simulations, followed by the diffusion coefficient, in turn followed by the advection and the proliferation rates (Fig. 5A,B). This analysis highlights the relevance of the proximalisation velocity to the dynamics of the electroporated cells and their progeny within the expanding limb during the time of regeneration.

### Model-predicted high expression of proximalisation factors enhances proximal patterning

An interesting question is whether increasing the initial expression levels of proximalisation factors such as Prod1 and Tig1 would further shift the cell density distributions toward more proximal positions. To test this hypothesis, we first ran simulations in which the initial conditions used in Results "Prod1 and Tig1 distal-to-proximal displacements can be described in terms of non-zero proximalisation velocities" for the Control, Prod1, and Tig1 conditions were replaced by Gaussian distributions. For each condition, we retained the same parameter values previously used in Results "Prod1 and Tig1 distal-to-proximal displacements can be described in terms of non-zero proximalisation velocities"—that is, the corresponding best-fitting values for diffusion coefficient, proliferation rate, advective velocity, and proximalisation velocity. These simulations revealed that doubling the area under the initial Gaussians broadened the resulting density profiles but did not change the distance between their peaks (Supplementary Fig. 5), suggesting that the proximalisation velocity does not depend on the initial density of electroporated cells.

In prior work, we showed that higher doses of Tig1 or Prod1 reduce cell proliferation by over 50%^33^, and that elevated Tig1 levels impair the invasive capacity of AL1 cells in vitro by approximately half^33^. To reflect this scenario, we re-simulated the Tig1 condition with both the proliferation rate and advective velocity reduced by 50%, while keeping the diffusion coefficient and proximalisation velocity unchanged. Under these conditions, the Tig1 density peak shifted further toward proximal positions, and the overall length of the regenerating domain decreased (Supplementary Fig. 6), consistent with our previous experimental observations^33^. Notably, in vivo, high Tig1 levels are also associated with additional morphological alterations that lie beyond the scope of our one-dimensional model.

## Discussion

Understanding how an organism such as the axolotl is able to regenerate limbs could push the boundaries of what is possible^39^. Upon amputation, progenitor cells at the stump can migrate over distances on the order of half a millimetre to reach the wound site, where they begin to form the blastema^40^, but how do they know where to go and where they are in the first place? Previous evidence shows that PROD1 and TIG1 act as chemical cues conferring proximal positional information in the limb. Overexpression of the respective genes during the early stages of axolotl limb regeneration induces a displacement of cells towards more proximal regions, a phenomenon known as proximalisation, suggesting that these genes play a key role in determining the positional identity of cells^20,22,25^^,^^26,28,32^^,^^33,41–43^. Although much experimental work has been done to uncover where the blueprint that orchestrates the orderly regrowth of missing tissue is encoded, there is currently no theoretical approach aimed at unravelling the spatio-temporal dynamics of the proximalisation process.

In this study, we quantitatively analysed displacement assays in which local electroporation of Prod1 or Tig1 onto the distal-most compartment of 4-day blastemas led to displacements of the labelled cells towards more proximal regions. Using Meandros, a Python-based image analysis script, we determined the linear density of distally electroporated cells and their progeny along the PD axis of the regenerating axolotl limb and estimated the mean and standard deviation from Gaussian fits (Figs. 2A,B and 3A–E). The temporal evolution of the mean confirmed previous reports: compared to controls, cells overexpressing Prod1 or Tig1 experience a shift towards proximal regions^28,32,33^. Notably, we observed that the standard deviation increases with time and is enhanced by Tig1 and Prod1 overexpression, suggesting that a diffusive phenomenon could partially explain the cellular shifts. Nevertheless, electroporated cells and their progenies were immersed in the expanding domain of the regenerating limb (Fig. 3A–C and Supplementary Fig. 2A–C). The anisotropic growth of the axolotl limb generates a distortion in the symmetry of the original linear density distributions (Fig. 3A–C). In parallel, we observed that the area under the curve of linear density increased in time, suggesting that electroporated cells were proliferating (Supplementary Fig. 2D–F). From both time courses we extracted the advection coefficients of tissue expansion and the proliferation rates. Noteworthy, the mean growth rates (day^−1^) for the control (0.093 ± 0.005), Tig1 (0.087 ± 0.005) and Prod1 (0.084 ± 0.004) (Table 1) are consistent with growth rate previously measured in regenerated limbs of axolotls of similar size^44^. Neither the expansion of the limbs nor the increase of the area under the curves of linear densities was affected by overexpressing Prod1 or Tig1 (Supplementary Fig. 2A–F). This probably reflects the fact that the doses used for these two factors were smaller than those used in previous reports, where defects and malformations in the regenerated tissue were observed, particularly in the distal parts^32,33^. A global dosage-effect probably underlies the absence of morphological defects and size reduction observed^32,33^. Here, the lower transgene dose used and the small number of transfected cells were likely insufficient to generate broad-scale effects. However, in whole-blastema Tig1-transfected samples, where higher transgene load and a wider distribution of Tig1 expression was achieved, significant size reduction, specially affecting distal structures, and other morphological alterations consistently occur^33^.

To disentangle proximalisation from the complexity of the axolotl limb regeneration response, we developed a minimal PDE-based mathematical model of electroporated cells and their progeny. Beginning with the seminal article by Alan Turing^45^, PDE-based mathematical models were proposed to understand pattern formation problems in biological contexts. This article, together with that of Alfred Gierer and Hans Meinhard^46^, exemplified the use of reaction–diffusion models that would become widespread in both development and regeneration. A notable example of this type of model was developed to understand the spatio-temporal distribution of skeletal elements in the developing chick limb^47^ and, more recently, the developing limb skeletal structures in mice and axolotl^48^ as well as the problem of morphogen scaling in the axolotl limb during regeneration^49,50^. In our study, we modelled both the domain expansion of the regenerating limb in which cells are immersed and the proximalisation phenomenon experienced by cells overexpressing Prod1 or Tig1 as advective processes, inspired by a large tradition of reaction–diffusion–advection mathematical models (see, e.g.,^34,51–53^.

By fitting the model to the spatio-temporal distribution of linear densities, we estimated diffusion coefficients and proximalisation velocities of electroporated cells and their progeny. Our results indicate that cells overexpressing Prod1 diffuse and move proximally more than those overexpressing Tig1, which in turn diffuse and move proximally faster than controls (Fig. 4B–D). This is the first study to predict diffusion coefficients and proximalisation velocities of cells within regenerating axolotl limbs, which range from 0.9 to 7.0 (${{10}^{3}\mu {m}^{2}}{day}^{-1}$) and 1.0 to 50.0 ($\mu m \, {day}^{-1}$), respectively. The lack of information on diffusion coefficients and proximalisation velocities of cells in regenerating tissues, let alone axolotls, makes it extremely difficult to compare our predictions with previous reports. However, diffusion coefficients and migration velocities of individual cells in different in vitro systems can be given as an upper bound. As examples of diffusion coefficients, human leukocytes and endothelial cells in agarose include values of approximately 8^54^ and 20^55^ (${{10}^{3}\mu {m}^{2}}{day}^{-1}$), respectively. In terms of migration rates, rat embryo fibroblasts show migration rates of approximately 700–900 $\mu m \, {day}^{-1}$^56^. The spontaneous mammary adenocarcinoma cell line CSML0 and the highly invasive rat glioma cell line BT4Cn exhibit cell speeds of 500–700 $\mu m \, {day}^{-1}$^57^. Mouse bone marrow-derived macrophages migrate at a speed of ~ 70 $\mu m \, {day}^{-1}$, increasing to ~ 600 $\mu m \, {day}^{-1}$ in response to Colony Stimulating Factor 1 (CFS-1)^58^. Our model predicts diffusion coefficients and migration rates that are significantly slower than those previously reported. This is probably because the aforementioned analyses were based on cells in culture, while our predictions derived from in vivo settings. Further, the electroporated cells and their progeny are not single cells, but clusters of cells that grow by cell proliferation, are exposed to the complex extracellular matrix in which they are embedded, and are subject to the cell–cell interactions that occur in vivo and are expected to dampen their movements.

**Fig. 6:**
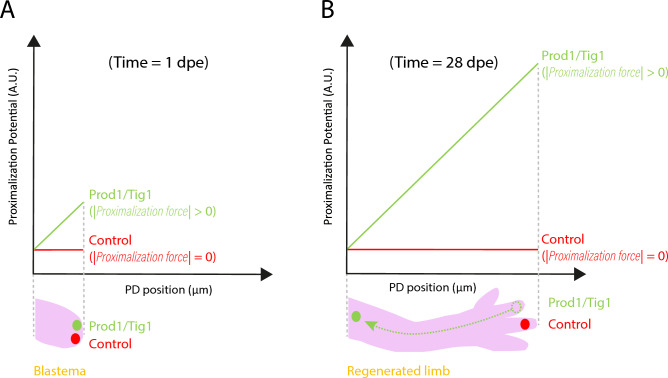
Proximalisation velocity is consistent with the proximalisation force derived from a spatially dependent potential. (**A**) Electroporated cells in the most distal region of the blastema at 1 day post electroporation (dpe): when only Gfp is electroporated (control, red), a spatially uniform proximalisation potential is generated. In contrast, electroporation of Tig1 or Prod1 (green) results in a non-uniform proximalisation potential. (**B**) Electroporated cells at 24 dpe: the proximalisation potential is constant for control cells (red), which motivates zero proximalisation force, forcing the cells to remain distally where they were electroporated. In contrast, the cells overexpressing Tig1 and Prod1 (green) experience a spatially dependent proximalisation potential that triggers a proximalisation force that induces their movement towards proximal regions.

The most important parameter of our model is the proximalisation velocity, as indicated by the sensitivity analysis (Fig. 5A,B). The model predicts that the proximalisation process results from a constant proximalisation velocity experienced by the electroporated cells and their progeny overexpressing factors such as Prod1 and Tig1. Using Smoluchowski’s theory, this velocity can be rewritten as a proximalisation force which, assuming constant mobility, should also be constant (Fig. 6A,B, Supplementary Information, Sect. 3.2). Assuming that the force is conservative, it can be derived from a proximalisation potential whose minimum is at the shoulder of the limb and which increases linearly along the PD axis (Fig. 6A,B, Supplementary Information, Sect. 3.3). Thus, according to our derivation, the electroporated cells and their progeny expressing high levels of Prod1 or Tig1 move proximally to minimise the proximalisation potential. Interestingly, this proximalisation potential can be associated with a chemotaxis-like process^59,60^, where the resulting proximalisation velocity can be written as the product of a chemotaxis strength and the negative gradient of the chemotactic species concentration gradient (Fig. 6A,B, Supplementary Information, Sect. 3.4). To explain our results, electroporated cells and their progeny highly expressing Prod1 or Tig1 should be capable of “sensing” gradients of chemotactic attractants, whose concentration should decrease linearly towards the distal end of the limb. Further, the chemotactic gradients or their strengths may differ between Prod1 and Tig1. What might this hypothetical chemotactic attractant be? A simple possibility could be Prod1 or Tig1 themselves since both show a decreasing expression along the PD axis (Fig. 6A,B,^29,33^, although precise quantification of both is lacking. While the nature of their interaction partners and their capacity for homotypic interaction are unknown, this remains an intriguing possibility.

An alternative hypothesis is that the increased presence of Tig1 and Prod1 membrane proteins modifies some physicochemical variable, such as surface tension, a hypothesis that has been repeatedly suggested in previous studies investigating Prod1^26,61,62^. A paradigmatic example consistent with this interpretation is the engulfment assay, where it has been suggested that the invasion of proximal cells into distal cells originates from differential adhesion due to increased levels of Prod1 at the cell surface^25^. Engulfment assays have also been conducted for Tig1, yielding similar results as Prod1^33^. Thus, transient distal expression of cells expressing high levels of Tig1 or Prod1 could lead to disruption of surface tension gradients and consequent cell migration to reduce these gradients^29^. Similarly, the gradient of proximalisation potential here proposed could reflect a gradient of stiffness. Interestingly, axolotl distal blastema cells are stiffer than proximal ones^63^, suggesting that proximalisation could be driven by negative durotaxis^64^.

### Limitations of our study and conclusions

In our study, the proximalisation process was modelled assuming that the electroporated cells and their progeny overexpressing factors like Prod1 and Tig1 are subjected to a constant proximalisation velocity. This velocity could encapsulate a more sophisticated mechanism involving feedback processes regulating positional memory while operating along the perpendicular anterior–posterior axis of the axolotl limb^65^. In general, the proximalisation velocity could depend on the regeneration stage as well as on the position along the PD axis. On the other hand, the population of electroporated cells could have an inherent heterogeneity, which could mean that each cell could have a unique proximalisation velocity. Thus, while the constant proximalisation velocity assumed by our minimal model fits the data presented here and satisfies Occam’s razor, it can also be conceived as an average over time, space and cellular identities.

In conclusion, this study presents a quantitative pipeline for analysing proximalisation in the limb during regeneration and introduces a continuous theoretical framework that portrays this phenomenon in terms of a proximalisation velocity. We hypothesise that proximalisation may be driven by a potential-driven force, consistent with a chemotaxis-derived process. Future studies are needed to further investigate the molecular and/or mechanical basis of proximalisation in vertebrates.

## Materials and methods

### Animal husbandry

Care of axolotls and all experimental protocols used in this study were approved by the United Kingdom Home Office and the State of Saxony, Germany. All methods were performed in compliance with the Animals -Scientific Procedures-Act 1986 (United Kingdom Home Office), and the laws and regulations of the State of Saxony, Germany. Axolotls (*A. mexicanum*) were obtained from Neil Hardy Aquatica (Croydon, UK) and from the axolotl Facility at Center for Regenerative Therapies Dresden (Germany). Axolotls of the leucistic (d/d) strain, of 4 cm snout-to-tail length, were used in all experiments. The animals were maintained in individual aquaria at 18–20 °C with a 12/12 day/night cycle, and were anaesthetised in 0.03% benzocaine (Sigma) prior to any surgical procedure or imaging. All methods are reported in accordance with ARRIVE guidelines.

### Blastema generation, plasmid electroporation and imaging of the regeneration timeline

Animal procedures were conducted as described^33^. Briefly, blastemas were generated by amputating the intact limb at the distal end of the upper arm (stylopod), followed by trimming of the jutted-out tip of the humerus. To prevent postoperative pain, animals were treated with a centrally acting analgesic for the next 24 h, by keeping them in shallow water containing 0.5 mg/l butorphanol tartrate (Alvegesic vet. 10 mg/ml, Selectavet). They were then returned to their individual holding tanks, where they were kept until the end of regeneration. At the 4th day of regeneration, the distal region of the blastema mesenchyme was transfected via microinjection and co-electroporation of Tig1 or Prod1 gene-containing plasmids and a reporter plasmid (pEGFP-N2 or pRFP-N2; Clontech), at a 1:3 molar ratio. Both the cloning strategy, electroporation protocol and experimental details were previously described^33^. Fluorescent and brightfield images were acquired at 1, 7, 12, 18, 24 days post-electroporation (dpe), using a stereomicroscope (Zeiss) to track the distribution of electroporated cells (Supplementary Fig. 1). Exposure times were generally maintained constant within and between conditions, only slightly adjusted, if necessary to capture sufficient fluorescent signal. During imaging, animals were consistently positioned in a similar orientation to ensure comparability across all samples and timepoints.

### Landmark setting

The cross-sectional plane created upon amputation, denominated as ‘amputation plane’, was defined at 1 dpe using the brightfield channel, and is morphologically defined by a decrease of epidermal tissue thickness and a disruption in mesenchymal mass. Its relative location was manually identified and defined conservatively across timepoints, setting the proximal boundary of the blastema/regenerate. The hand (autopod) segment was defined from a section crossing the point where the proximal radius and ulna bone epiphyses are at the closest point, and extends until the distal end of the computed PD axis. The ‘lower arm’ (zeugopod) segment is limited by the later boundary and by the distal tip of the humerus, which in turn simultaneously defines the distal end of the ‘upper arm’ (stylopod) segment. Segment boundaries were defined manually by the same user, across samples and between timepoints. These landmarks appear as vertical regions in Fig. 4B–D.

### Image analysis to determine the cell density profile from fluorescent microscopy images

To obtain the cell density profiles along the PD axis (Posterior-Distal), we used our software Meandros. The microscopy images were individually loaded for each time point. The region corresponding to the axolotl’s limb (ROI) was obtained using the ROI detection tool, followed by manual fine-tuning to ensure that the ROI contained the entire limb to be analysed. Using the GUI, the PD axis was traced so that it passed through pre-established bony landmarks: the elbow joint, wrist joint. This ensured that all PD axes for all replicates were systematically established.

A lower intensity threshold was set to avoid background noise and filter only positive intensity. In cases where artifacts were identified, the exclude area tool was used to exclude regions of false-positive intensity signal. To distinguish true intensity signal from false positives, we relied on our extensive familiarity with the image datasets, which allowed us to recognize and therefore discard artefactual autofluorescence. More importantly, since the trajectories of electroporated signals were tracked over time, it was possible to anticipate their likely spatial paths and exclude intensity signals that appeared in locations inconsistent with plausible trajectories. This temporal continuity provided an additional criterion to discard spurious intensity signals that could not have originated from the electroporated region. The profiles were normalized according to the number of positive pixels (above the threshold) perpendicular to the point of the PD axis relative to the maximum found along the PD axis.

${\rho }_{i}=\frac{{f}_{i}}{{f}_{max}}$, where *i* represents the position along the PD axis, and, $0<i<L(t)$

Mean and error of all replicates for each condition in Fig. 3A–E were calculated using the stats module of SciPy^66^. The signal for each time point was normalized by the integral under the curve at time 1. This way, the growth of the area under the curve is expressed in multiples of the area of the initial condition.

### Mathematical modelling of proximalisation

#### Model implementation

We numerically implemented the mathematical model described in Results "Mathematical model of proximalisation in the regenerating axolotl limb".

When numerically solving Eq. (7) we made a change of variables to a non-growing domain (see, for example^51,52^). The chosen variables were:

8 $$\delta =\frac{x}{{{L}_{0}e}^{at}}, \delta \in \left[\text{0,1}\right]$$

9 $$\theta =t$$

Equation (7) written in these new variables is:

10 $$\frac{\partial \rho }{\partial \theta } = \frac{D}{{{{(L}_{0}e}^{a\theta })}^{2}}\frac{{\partial }^{2}\rho }{{\partial \delta }^{2}} + (r - a)\rho + \frac{v_p}{{L}_{0}{e}^{a\theta }}\frac{\partial \rho }{\partial \delta }$$

And the new non-growing boundary conditions are the following:

11 $$\frac{\partial {\rho }}{\partial \delta }\left(\delta =1,\theta \right)=0$$

12 $$\frac{\partial {\rho }}{\partial \delta }\left(\delta =0,\theta \right)=0$$

This change of variables had two main advantages, the first one being that the new domain size is fixed (going from 0 to 1) and secondly that Eq. (10) now has one less advective term than Eq. (7).

The upwind method was used to simulate Eq. (10) because it is more stable than using the finite difference method. A similar implementation as the one proposed in^67^ and^68^ was used. This method is applicable to equations with the following shape:

13 $$\frac{{\rho }_{i}^{n+1}-{\rho }_{i}^{n}}{\Delta \theta }+\frac{{(\rho G)}_{i+\frac{1}{2}}-{(\rho G)}_{i-\frac{1}{2}}}{\Delta \delta }=0$$

where G is an arbitrary function of $\rho ,\delta ,\theta ,\frac{\partial \rho }{\partial \delta }$. In our implementation G took the form:

14 $$G=\frac{-D}{{{{(L}_{0}e}^{a\theta })}^{2}\rho }\frac{\partial \rho }{\partial \delta }-\frac{v_p}{{L}_{0}{e}^{a\theta }}$$

To calculate ${(\rho G)}_{i+\frac{1}{2}}$ and ${(\rho G)}_{i-\frac{1}{2}}$, we followed the rule:

15 $$(\rho G)_{i+\frac{1}{2}} = \rho_{i} \frac{G_{i} + G_{i+1}}{2} \quad \text{if} \quad \frac{G_{i} + G_{i+1}}{2} > 0$$

16 $$(\rho G)_{i+\frac{1}{2}} = \rho_{i+1} \frac{G_{i} + G_{i+1}}{2} \quad \text{if} \quad \frac{G_{i} + G_{i+1}}{2} < 0$$

This scheme has a Courant–Friedrichs–Lewy (CFL) condition given by $2max\left|G\right|\frac{\Delta \theta }{\Delta \delta }$. This condition ensures that information does not propagate farther than one spatial grid cell during a single time step, which is crucial for maintaining numerical stability. We used an *n*_*0*_ = 200 nodes array, with *x* step $dx=\frac{1}{{n}_{0}}$ y $dt=\frac{{dx}^{2}}{3D}$

#### Estimation of the advection rate parameter *a*

To estimate the value of the parameter *a* in Eq. (7), we fitted an exponential to the length of the regenerating tissue *L(t)* (Supplementary Fig. 2A–C). The *L(t*) was calculated as the arc length of the PD axis $s \approx \sum_{i=1}^{n} \sqrt{\Delta x_i^2 + \Delta y_i^2}$ for all replicates of each experimental condition. *L(t)* was then adjusted over time to a target function $c.exp(a.t)$ using the curve_fit tool from the optimize module of the Scipy library^66^. The standard deviation of the parameters was calculated and confidence intervals were determined using a Student’s t-distribution with *N* − 1 degrees of freedom, where *N* is equal to the number of replicates. The calculation of confidence intervals is given by $CI=a\pm {t}_{\frac{\alpha }{2}}.std$, where *a* is the advection parameter, ${t}_{\frac{\alpha }{2}}$ is the critical value from the Student’s *t*-distribution for a significance level $\alpha$ and *N* − 1 degrees of freedom, and *std* is the standard deviation.

#### Estimation of the proliferation rate parameter *r*

In order to estimate the parameter *r* in Eq. (7), we calculated the area under the curve of cell density profiles for all replicates individually using the numerical integration tool simps from the integrate module of Scipy. The Areas Under the Curve (*AUC*s) were then normalized by the area of the initial condition: $AUC(t) = \frac{I(t)}{I_0}$ where *I(t)* represents the area at time *t* and *I*_*0*_ is the area of the initial condition. The normalized values $Mass(t)$ were adjusted over time to a target function $c.exp(r.t)$ and confidence intervals were obtained using the same procedure as for the parameter *a*.

#### Posterior distributions of the parameters ***v***_***p***_*** and D*** using a Bayesian inference framework.

To estimate the parameters *v*_*p*_ and *D*, we used the formalism of Bayesian Inference and obtained the posterior distribution of the parameters. Within the context of Bayes’ Theorem, we have:

17 $$P(\theta | Y) = \frac{P(Y | \theta) \cdot P(\theta)}{P(Y)} = \frac{P(Y | \theta) \cdot P(\theta)}{P(Y | \theta) \cdot P(\theta)}$$

where ${Y}^{t}=\{{y}_{0},{y}_{1},{y}_{2},...,{y}_{N}\}$ are the points belonging to the density profile at time t for each $t\in \{\text{6,12,18,24}\}$, and $\theta$ represents the set of parameters.

Assuming a Normal error of the experimental data with $\mu (\theta )$ and standard deviation $\sigma$, likelihood is:

18 $$P(y_{i}^{t} | \theta) = \mathcal{N}(y_{i}^{t} | \mu(\theta), \sigma)$$

And assuming that the observations ${y}_{i}^{t}$ are independent.

19 $$P^{t}(Y^{t} \mid \theta) = \prod_{i=0}^{N} \mathcal{N}(y_{i}^{t} \mid \mu(\theta), \sigma) = \prod_{i=0}^{N} \mathcal{N}(y_{i}^{t} \mid \mu(v, D), \sigma)$$

where $$\theta =\{v,D,\sigma \}$$

The posterior distribution over the parameters at time t + 1 is:

20 $$P^{t+1}(v, D, \sigma \mid Y^{t+1}) = \frac{\prod_{i=0}^{N} \mathcal{N}(y_{i}^{t+1} \mid \mu(v_j, D_k), \sigma_l) \cdot P^{t}(v_j, D_k, \sigma_l)}{\sum_{v, D, \sigma} \prod_{i=0}^{N} \mathcal{N}(y_{i}^{t+1} \mid \mu(v_j, D_k), \sigma_l) \cdot P^{t}(v_j, D_k, \sigma_l)}$$

As a *prior distribution* ${P}^{0}(v,D,\sigma )$ a uniform distribution in the parameter space v, D, $\sigma$ was used. A matrix of dimensions ${N}^{3}$ with *N* = *100* for all possible combinations of parameters was constructed, and (4) was iteratively calculated. The marginal distributions shown in Fig. 4B–D were obtained by numerical integration of the posterior distribution.

21 $${P}_{\theta =x}({x}_{i})={\sum }_{j}{\sum }_{k}P({\theta }_{i,j,k})$$

To smooth the results due to the discretization of the explored parameter space, spline interpolation was applied using the interp2d tool from Scipy^69^.

To avoid overflow problems in the computational calculation of Eqs. (20) and (21), the "log-sum-exp trick" was applied to calculate a relative posterior within the range [0,1]^70^.

22 $${{P}_{\text{relative}}}^{t}({Y}^{t} \mid \theta) = \frac{e^{\log(P^{t}({Y}^{t} \mid \theta))}}{e^{\max(\log(P^{t}({Y}^{t} \mid \theta))}} = e^{\log(P^{t}({Y}^{t} \mid \theta)) - \max(\log(P^{t}({Y}^{t} \mid \theta)))}$$

As shown in Results "Image Analysis allows quantification of the proximalisation effect driven by Prod1 & Tig1", our experimental data consist of individual distributions of cell density ${\rho }^{data}(x,t)$ averaged for each condition (Control, Tig1 and Prod1). Here, the different measurements were acquired at time points 1, 6, 12, 18 and 24 dpe. We used as initial conditions the quantified density profile at time 1 dpe of each experiment. We assumed that the observations ${\rho }^{data}(x,t)$ are noisy versions of the model-predicted density ${\rho }^{model}$. We assumed the errors being additive, independent and normally distributed with variance ${\sigma }^{2}$, enabling us to the model the error as:

23 $${\rho }^{data}({x}_{i},{t}_{j})={\rho }^{model}({x}_{i},{t}_{j})+\varepsilon ,\varepsilon \sim N(0,\sigma )$$

We used a non-informative uniform prior in the 3D parameter space given by *v*_*p*_, *D* and $\sigma$, constructed a matrix of dimensions ${N}^{3}$ for the parameters and calculated the posterior distributions *p*(*v*_*p*_, *D*, $\sigma$) across the entire parameter range, where *N* = 100. Finally, we obtained the marginal distributions *p*(*v*_*p*_), *p*(*D*), *p*($\sigma$) as well as the mean and standard deviation of each parameter.

#### Validation of Bayesian Inference implementation

To validate our Bayesian inference implementation described in Results "Prod1 and Tig1 distal-to-proximal displacements can be described in terms of non-zero proximalisation velocities", we generated synthetic noisy data to test its ability to recover the true parameter values. Cell density profiles were generated using the RDA model with known parameters within the range ${v}_{p}({day}^{-1})=(\text{0.0005,0.05})$, $D({day}^{-1})=(\text{0.00005,0.005})$ and $\sigma =(\text{0.0001,0.01})$. The posterior distributions and marginal distributions of the parameters were obtained following the same approach described in Sect.  4.3.3. These results confirm that our Bayesian inference implementation successfully identifies the optimal parameter values for synthetic data generated by the RDA model within the specified study ranges.

#### Posterior distributions of the parameters obtained by using pyABC

PyABC (Approximate Bayesian Computation in Python) is a Python library for performing Approximate Bayesian Computation (ABC), which is used to estimate parameters of complex models by comparing simulated and observed data without requiring an explicit likelihood function. The implementation of pyABC fundamentally requires specifying the prior distributions of the parameters and a distance function to be minimized^36^. Since there is no prior knowledge about the parameter values, we used uniform priors for the parameters *v*_*p*_ and *D*, limiting the analysis within the appropriate bounds for each condition. We defined a distance function in the form: $\frac{\sum_{i}^{N} (y_{i} - f(x_{i}))^2}{\sigma^2}$, where ${y}_{i}$ are the experimental values of *cell density* at position *i* of PD axis (which are assumed independent and normally distributed), $f({x}_{i})$ are the corresponding values generated by the RDA model and $\sigma$ is the sample variance by condition. Here, we used the $\sigma$ values from previous analysis (see Sect.  4.3.3): Control (0.006367), Tig1 (0.005426), Prod1 (0.005803). The following model params were setted: *population_size* = *100, max_nr_population* = *20 y minimum_epsilon* = *0.1 (acceptance threshold)*. Results are shown in Supplementary Fig. 4A–C.

## Supplementary Information

Supplementary Information.

## Data Availability

The data corresponding to Figs. 1, 2, 3A–E, 4B–D and 5A,B as well as Supplementary Fig. 2A–F, Supplementary Fig. 3A,B and Supplementary Fig. 4 is available in 10.5281/zenodo.15365665. The Meandros software (see more details in the "Introduction" of Supplementary Text) can be found in 10.5281/zenodo.15036317.

## Code availability

The codes corresponding to the model, simulations and calculations of the parameter posterior distributions described in "Mathematical modelling of proximalisation" can be found in 10.5281/zenodo.15365665. Further information and requests for data should be directed to Osvaldo Chara (Osvaldo.Chara@nottingham.ac.uk).
