# Supplementary material for: Modeling proximalisation in axolotl limb regeneration
Source: Sci Rep. 2025 Jul 24;15:26839. doi: 10.1038/s41598-025-10527-8 (PMC12290015; doi:10.1038/s41598-025-10527-8)
Supplement: Supplementary file 1 — Supplementary Information. [file 41598_2025_10527_MOESM1_ESM.pdf]

Supplementary Information:

Modeling Proximalisation in Axolotl Limb Regeneration

Hernán Arce[1], Alberto Sebastián Ceccarelli[2], Rodrigo Carlos Córdoba[1], Ana Catarina Rodrigues Oliveira[4], Maximina Hee Yun[3,4,5] & Osvaldo Chara[1,2]*

[1] Instituto de Tecnología, Universidad Argentina de la Empresa, Buenos Aires, Argentina.
[2] School of Biosciences, University of Nottingham, Sutton Bonington Campus, Nottingham LE12 5RD, UK
[3] Chinese Institutes for Medical Research, Beijing, China
[4] Technische Universität Dresden, CRTD Center for Regenerative Therapies Dresden, Germany
[5] Physics of Life Excellence Cluster Dresden, Germany

*Corresponding author: osvaldo.chara@nottingham.ac.uk

Content

1. **Supplementary figures**……………………………………………………………………...3
2. **Meandros: computational tool for image segmentation in curved tissues**………………9

    2.1. Region of Interest (ROI)...10

        2.1.1 Meandros Training Pipeline (MTP) - Training and validation……………….10

    2.2 Thresholding and Area exclusion……………………………………………………13

    2.3 Axis detection……………………………………………………………………...14

    2.4 Amputation Plane……………………………………………………………..…...17

    2.5 Landmarks………………………………………………………………….……...17

    2.6 Profiling module………………………………………………………..…………17

3. **Reaction-Diffusion-Advection theory of proximalisation in the axolotl regenerating limb**…………………………………………………………………………….…...18

    3.1 proximalisation as an advective process………………………………….…….18

    3.2 proximalisation as a force…………………………………………………….……...19

    3.3 proximalisation as a potential………………………………………………….…….19

    3.4 proximalisation velocity as a Chemotaxis-driven process…………………….…....19

4. **References**……………………………………………………………………….……...21

1. Supplementary figures

Supp. Fig. 1. Schematic overview of the displacement assay protocol.

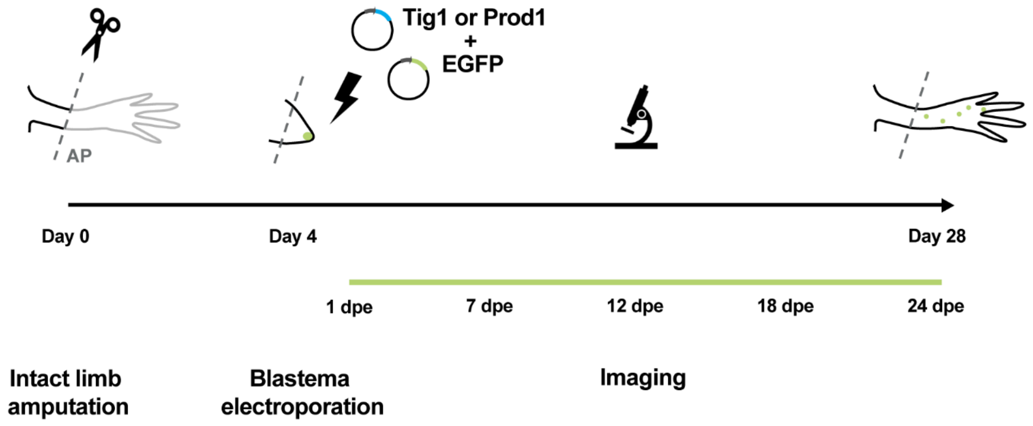

Supp. Fig. 1. An amputation is performed at the level of the distal upper arm (stylopod), from where a blastema will emerge. After 4 days, the distal early-staged blastema tip is electroporated with either Gfp alone, Tig1+Gfp or Prod1+Gfp plasmid combinations. Microscopic imaging is performed at 1, 7, 12, 18 and 24 days post electroporation to capture the position of cells within the regenerating limb, during regeneration. AP -amputation plane, dpe – days post electroporation.

Supp. Fig. 2. Exponential-like limb growth and cell density Area Under the Curve (*AUC*) enable estimation of proliferation rate (*r*) and advection rate (*a*).

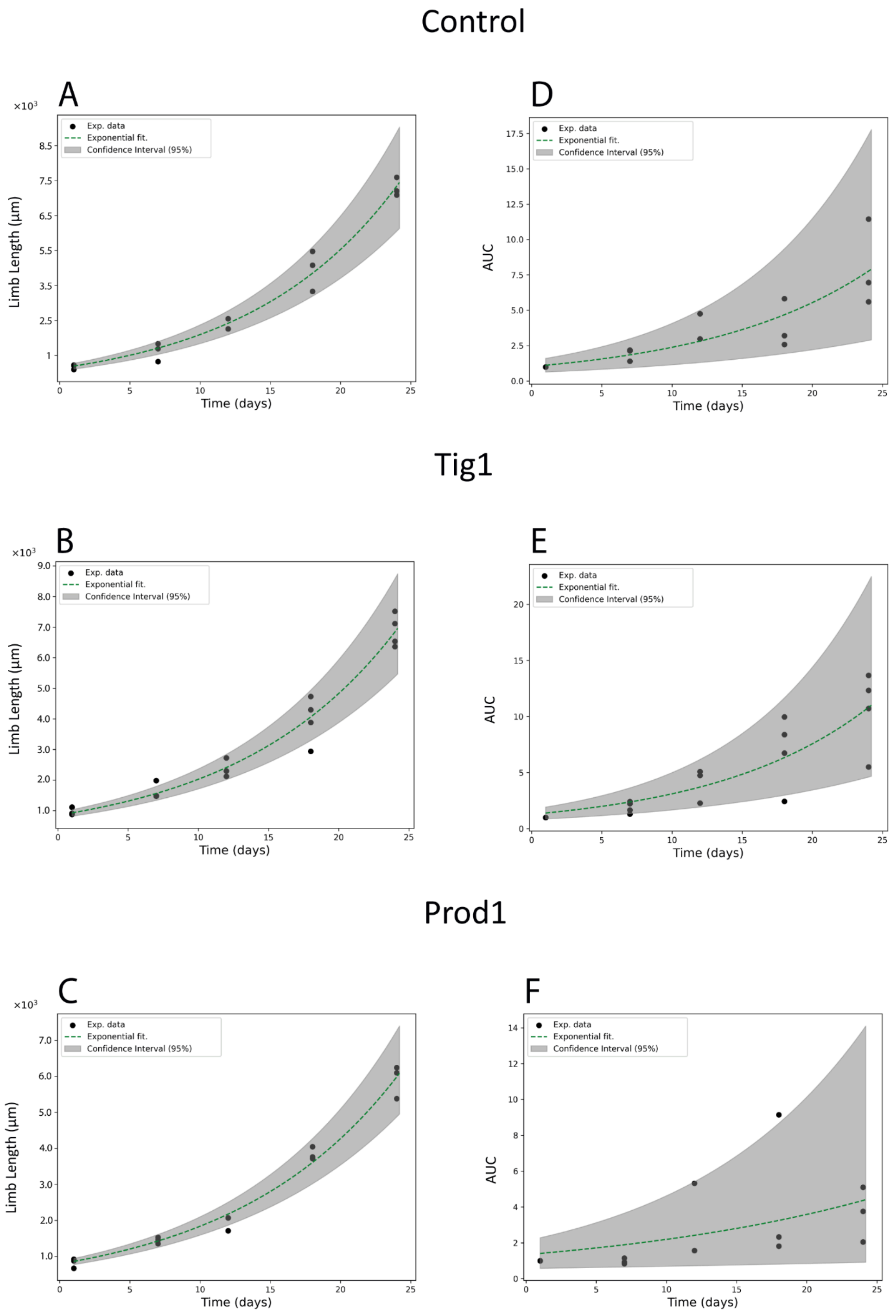

Supp. Fig. 2. A-C) Exponential fitted to the axolotl limb length during regeneration for the control (A), Tig1 (B) and Prod1 (C). **D-F)** Exponential fitted to the AUC of cell density in the axolotl limbs during regeneration for the control (D), Tig1 (E) and Prod1 (F). Time is expressed in days post electroporation (dpe). Control (n=3), Tig1 (n=4), Prod1 (n=3). The solid circles represent the experimental data, the dashed line represents the fitted curve, and the shaded area represents the region between the curves formed by the upper and lower ends of the confidence interval of the exponential parameters (95% confidence interval). Best-fitting values of *r* and *a* in Table 1.

Supp. Fig. 3. The Bayesian approach enables accurate prediction of artificial data with known parameters.

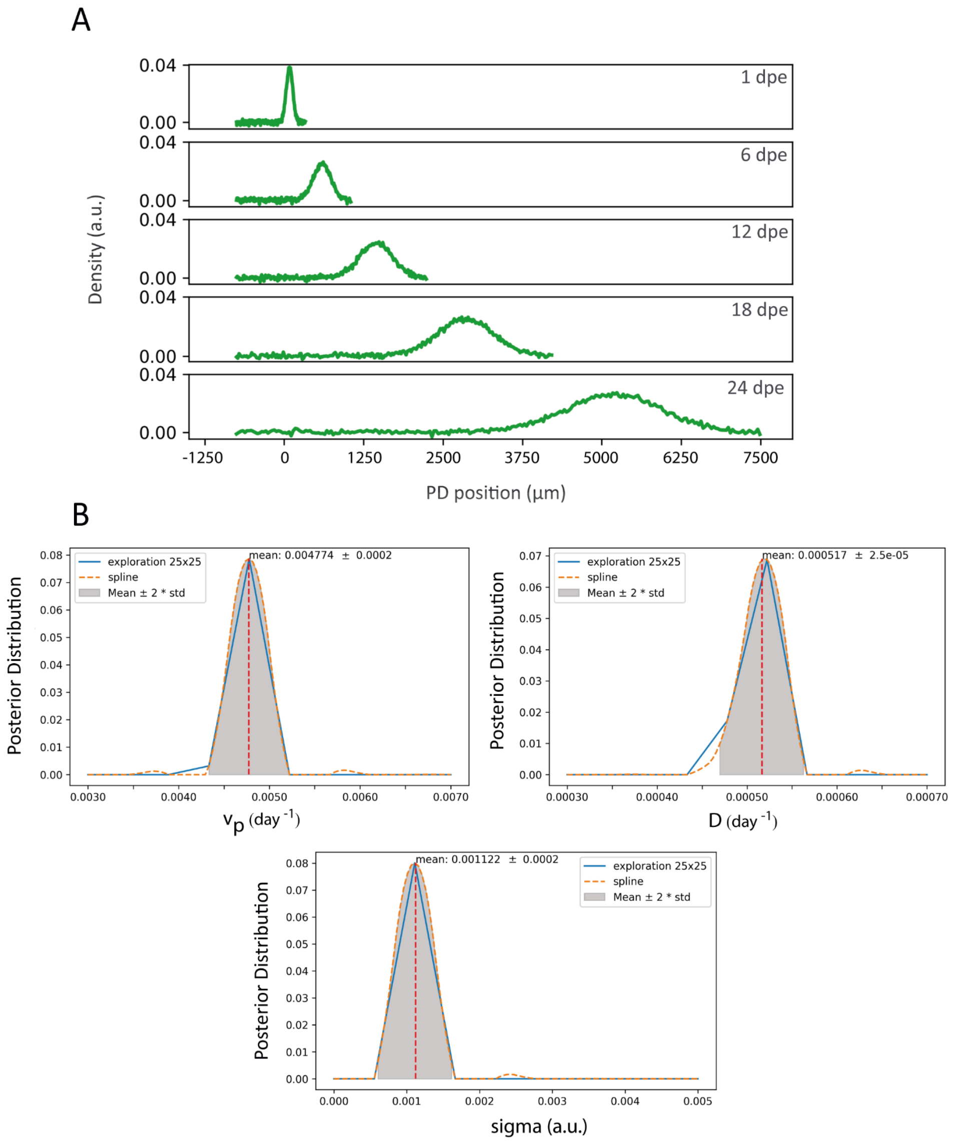

Supp. Fig. 3. A) Artificial data with known values of parameters v_p, D, and sample deviation generated using the proximalisation model. The solid green line represents cell density along the proximal-distal axis for the time series at 1, 7, 12, 18, and 24 days post-electroporation (dpe). **B)** Marginals on parameters v_p, D, and sample deviation (sigma). The solid blue line shows the distribution for a 100 by 100 grid exploration of the parameter space. The dashed orange line is smoothed with a quadratic spline. The dashed red line represents the mean of the distribution, and the shaded area corresponds to two standard deviations. This demonstrates that the algorithm is capable of predicting the values of the parameters that best fit the artificial data. The values used in this simulation were: v_p = 0.005 dpe^{-1}, D = 0.0005 dpe^{-1}, sigma = 0.001 where the parameter values are normalised by the initial tissue length L_0.

Supp. Fig. 4. Optimization of v_p and D parameters using the pyABC tool

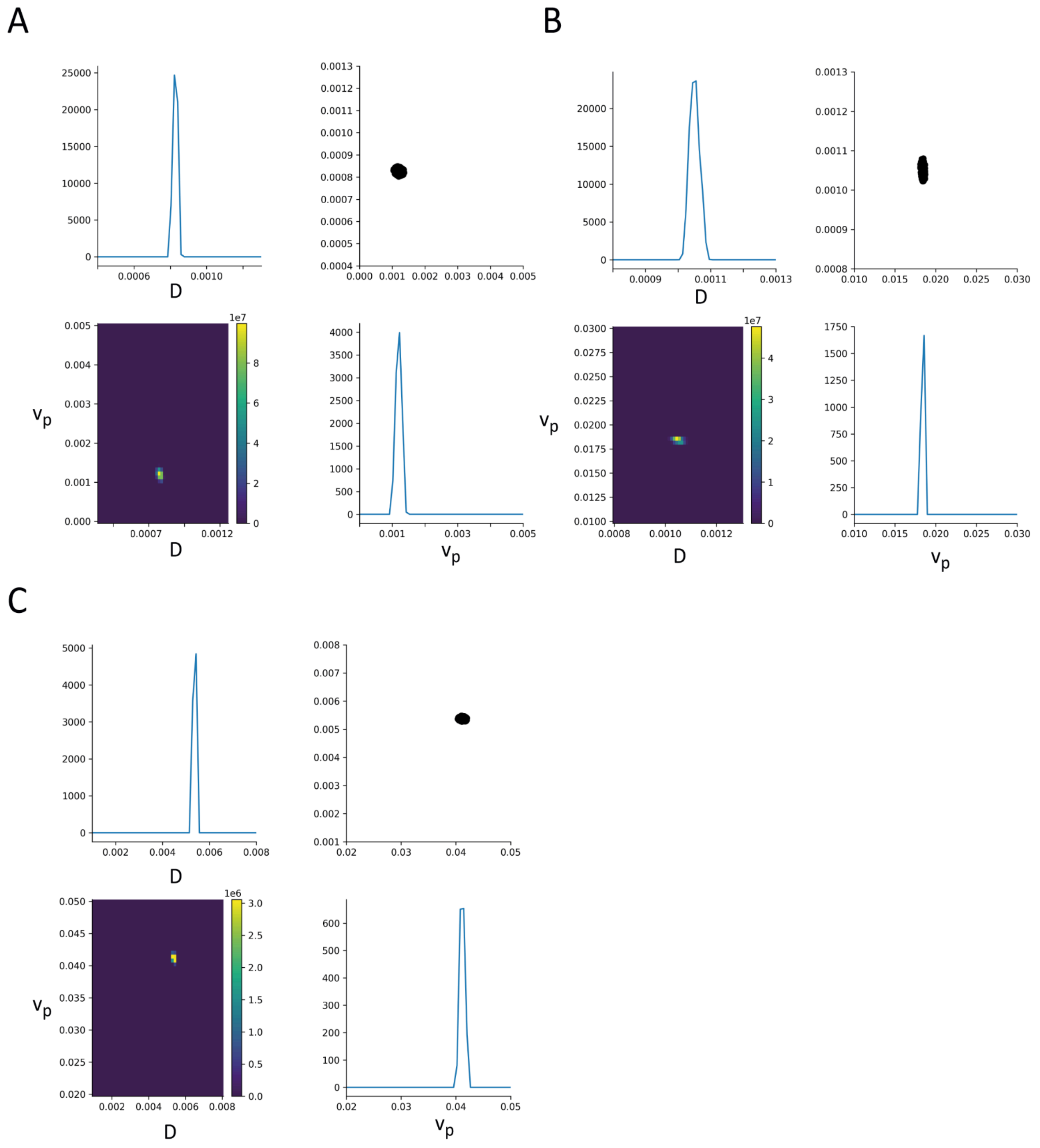

Supp. Fig. 4. A) Parameter fitting for the control condition using pyABC tool. On the diagonals of the matrices, marginal distributions for D and v_p are shown. Bottom left: posterior probabilities $p(v_p, D)$. Top right: Final dispersion of particles used in Sequential Monte Carlo simulation: each particle represents a candidate parameter set for the model, and it is updated through resampling using a Gaussian kernel (see pyABC documentation). **B)** Params fitting for the Tig1 condition using pyABC tool.. Same as A) **C)** Results of the pyABC analysis for the Prod1 condition. Same as A). Note that the values of v_p and D are dimensionless according to the change of variables described in Section 4.3. To recover the parameter values in the corresponding units, the following transformations are necessary: $v_p = v_p' L_0$ and $D = D' L_0^2$.

Supp. Fig. 5. Increasing the initial density of cells does not affect proximalisation dynamics when model parameters are not altered.

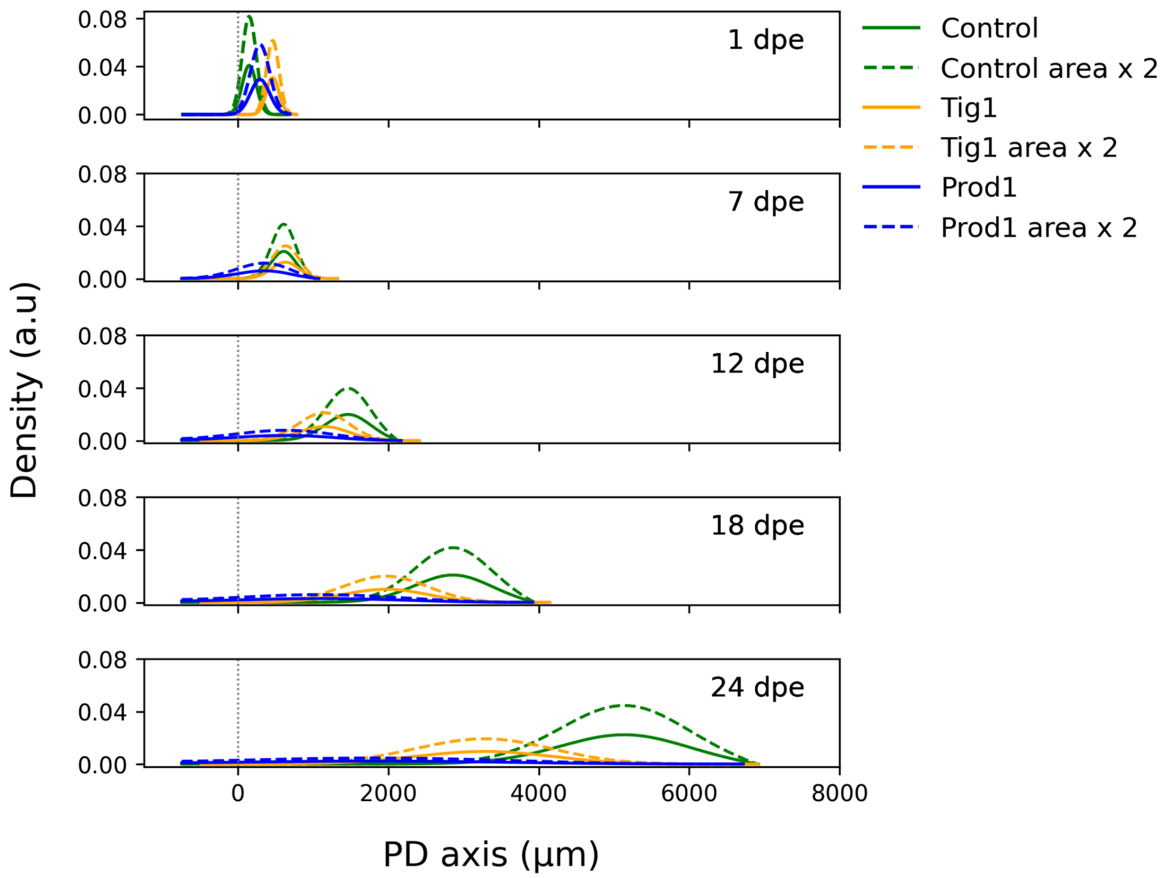

Supp. Fig. 5. Six simulations are shown: for each condition (Control, Tig1, and Prod1), two initial conditions were used—a Gaussian distribution parametrized from the corresponding experimental initial density profile, and a Gaussian with double the area under the curve. The Gaussians were fitted to the experimental distribution at 1 dpe for Control, Tig1 and Prod1. The model parameters (diffusion coefficient, proliferation rate, advective velocity, and proximalisation velocity) were set to the best-fitting values previously determined for each experimental condition (see Section 2.4). Panels show the initial density profiles (top) and the evolution of the distributions at four subsequent time points (remaining panels). Doubling the area under the initial Gaussian broadens the resulting density distributions but does not affect the position of the density peaks, indicating that proximalisation velocity is independent of the initial density magnitude.

Supp. Fig. 6. Effects of increased initial density and reduced proliferation and advective velocity on Tig1-driven cell density dynamics compared to Control.

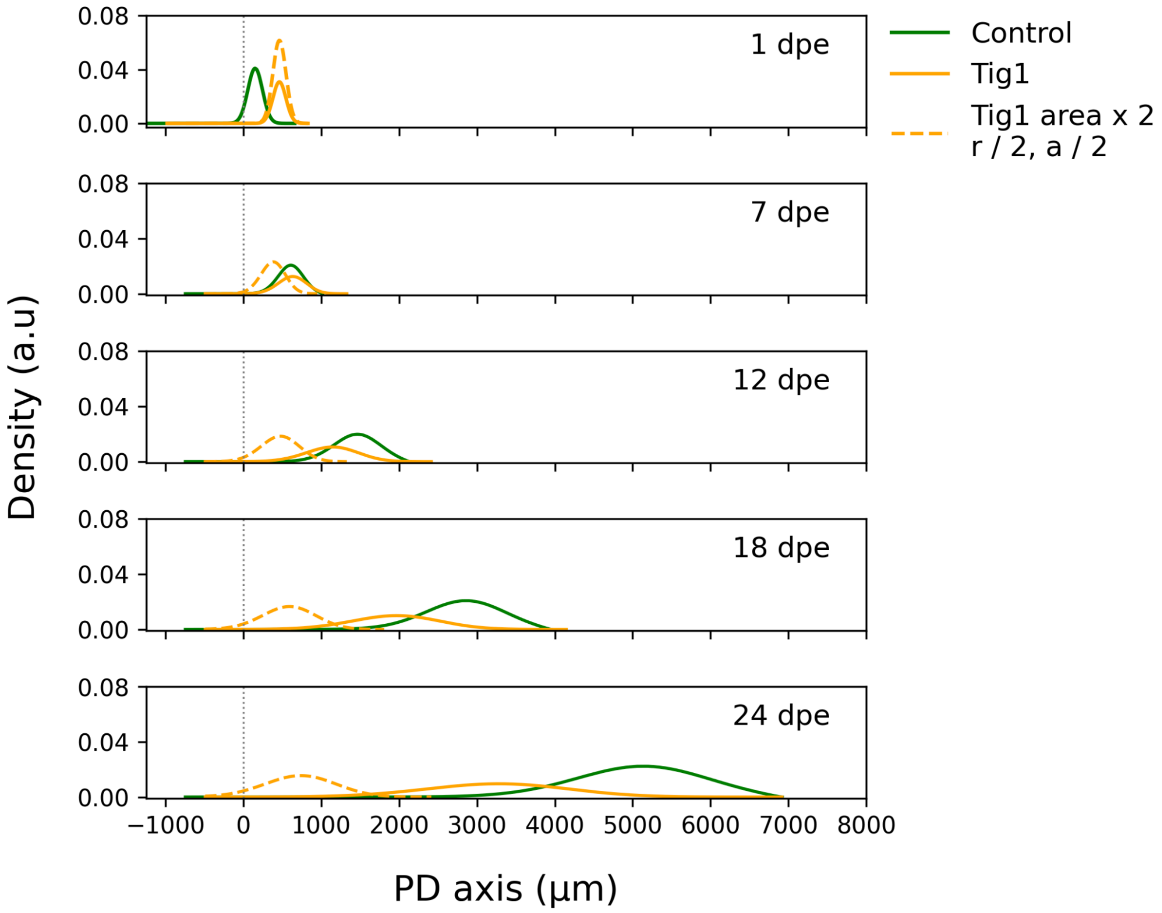

Supp. Fig. 6. Five panels show the initial cell density profile (top) followed by four subsequent time points. Three simulations are presented: Control, Tig1, and a modified Tig1 scenario. The Control and standard Tig1 simulations start from Gaussian initial conditions parametrized from experimental cell density distribution at 1 dpe, using their respective best-fitting values of the model parameters as described in Section 2.4. The modified Tig1 simulation also starts with a Gaussian initial condition but with double the area under the curve compared to the standard Tig1. In this scenario, the proliferation rate and advective velocity are reduced by 50% relative to the Tig1 best-fitting values, while the diffusion coefficient and proximalisation velocity remain unchanged. In this condition, Tig1 results in a further proximal shift of the density peak and a reduction in domain length.

2. Meandros: computational tool for image segmentation in curved tissues

To analyse the spatial profiles of the cellular densities of electroporated cells and their progeny in the axolotl limbs, we created Meandros [1]. This computational tool allows the quantification of density profiles in curved tissues, such as axolotl limbs. Meandros is a customizable, Python-based user-friendly, robust and capable of addressing different tissue geometries and cell density patterns. The user can interact with Meandros through command line or a Graphical User Interface (GUI) (Fig. S1).

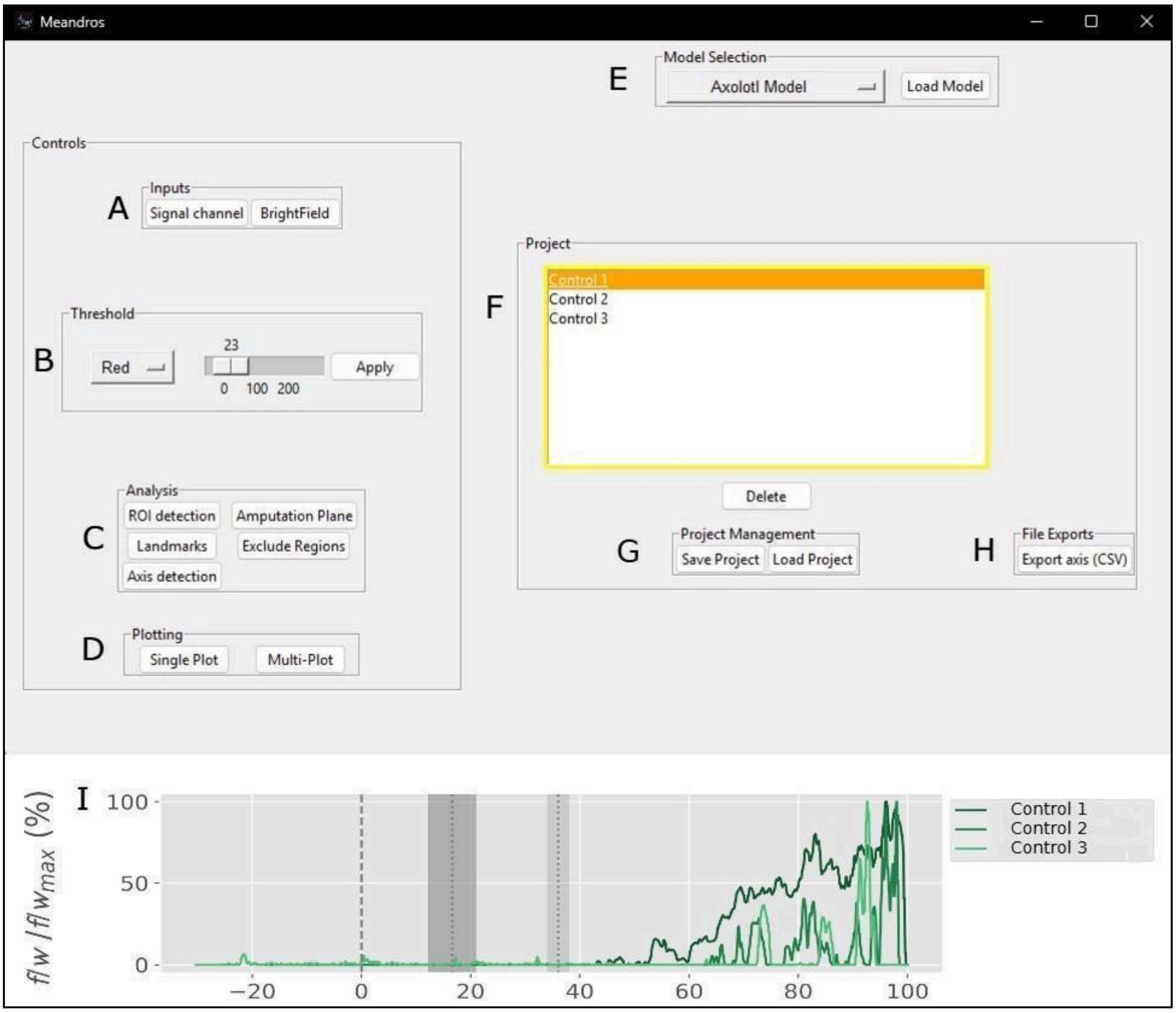

Figure S1. Meandros's Graphical User Interface (GUI). **A)** The input options allow the user to select and upload either a brightfield image or a signal channel image. **B)** The thresholding functionality enables users to set a threshold value while selecting the channels, depending on the biomarker being used. **C)** Analysis features. The "ROI Detection" button outputs the predicted region of interest (ROI) from the model. The "Amputation Plane" button allows users to draw and define the amputation plane in the image. Additional features include "Landmarks" and "Exclude Regions," which enable users to define or exclude specific regions of interest. The "Axis detection" button automatically detects the PD axis for the image, which can also be manually adjusted by the user if needed. **D)** Statistical features. The "Single Plot" option generates plots of fluorescence intensity, while the "Multi-Plot" feature provides a profile analysis of a group of selected images. **E)** Model selection, where the user can choose which model would be preferred to detect the region of interest (ROI). **F)** This allows the user to control all the files that are currently being used **G)** project exportation, importation, and saving **H)** export of the axis coordinates after being predicted by Meandros. **I)** Profile output of Meandros using the profiling tool explained in 1.6.

2.1. Region of Interest (ROI)

Meandros recognises the ROI by using a deep-learning strategy (Fig. S2), based on the Mask R-CNN architecture [2] using ResNet101 [3] as the convolutional neural network (CNN) backbone. ResNet101 is a specialized architecture for segmentation of high-complex images. Convolutional Neural Networks (CNNs) are a type of neural network that are particularly well-suited to image analysis tasks. The software was implemented in Python, utilizing Tensorflow and Keras as the main libraries for implementation[4].

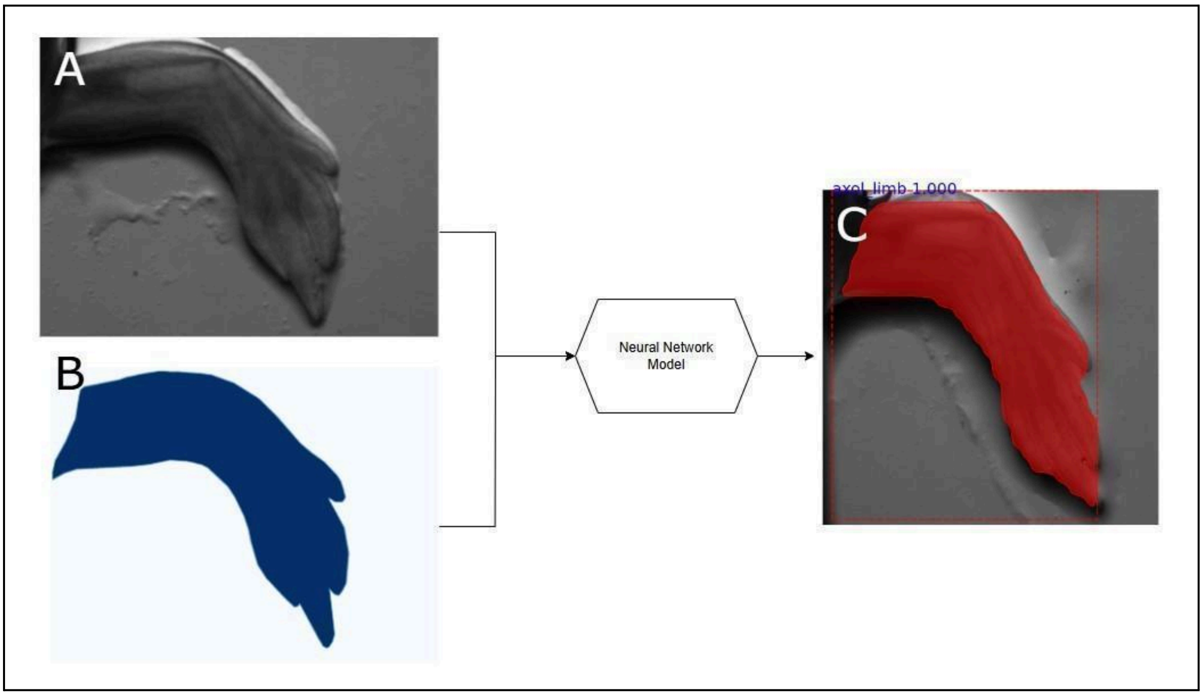

Figure S2. Flowchart illustrates the deep-learning strategy employed by Meandros to identify the Region of Interest (ROI) within axolotl limbs. A) Image of an axolotl limb. **B)** The ROI, annotated using the online tool VIA [5], is provided alongside the original image as input to the neural network model. **C)** The detected ROI is highlighted as a red overlay mask superimposed on the axolotl limb. Two classes were created: "axol_limb" and "axol_early_limb". The first corresponds to images where the limb is fully regenerated with all the fingers completely grown, while the second corresponds to limbs that are not fully regenerated. The hyperparameters that were modified for the model include the confidence level, set to 0.90, and the steps per epoch and epochs, both set to 100. The neural network was trained to classify the image between the two stages and detect the region of interest (ROI).

2.1.1 Meandros Training Pipeline (MTP) - Training and validation

The Meandros Training Pipeline (MTP) is a framework that utilizes deep learning techniques to analyse and interpret imaging data (Fig. S3*)*. This framework was built on the Mask R-CNN architecture (see previous Section), which allows for the efficient and accurate analysis of large amounts of imaging data. The MTP was trained using a pool of 346 manually annotated images, by using the brightfield channel.

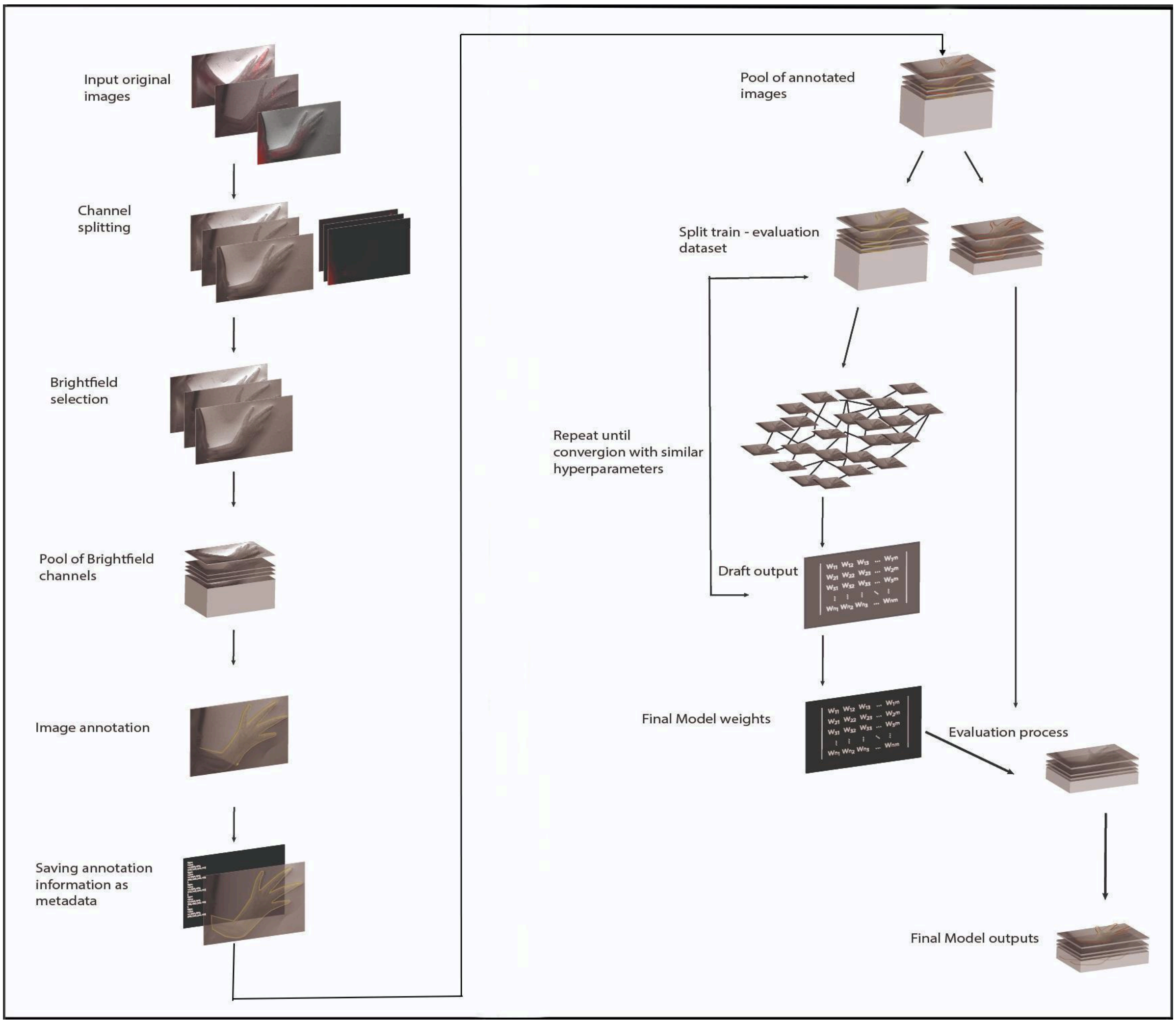

Figure S3. Meandros Training Pipeline (MTP). The brightfield channel was extracted and separated from the raw images. This collection of images was manually annotated, and the metadata was updated using the VGG Image Annotator software. The annotated images were then processed by a train-evaluation splitting algorithm, which randomly assigned 80% of the dataset to the training set and 20% to the evaluation set. The training dataset was used as input for the Meandros Neural Network, which underwent iterative training with random hyperparameters until the optimal model scenario was achieved. The final model weights were saved in an H5 file and were used to evaluate the model's performance on the evaluation dataset. When the accuracy exceeded 90%, the model was saved and integrated into Meandros.

The image dataset was partitioned into training and validation sets in a 70-30 split ratio. Subsequently, the pipeline initiated the first phase of training, exclusively focusing on the head layers of the neural network. During this phase, the model initialized its weights using the pre-trained weights from MS COCO [6] as a reference. Following this initial training phase, the first pre-trained model was generated based on the images.

The first training phase was followed by a second round of training, using the initial pre-trained model as a starting point, with no restrictions on layer freezing. This final training phase encompassed all layers of the network. At the end of this stage (Fig. S4), the resulting model was validated against the validation dataset, predicting the regions of interest within each image.

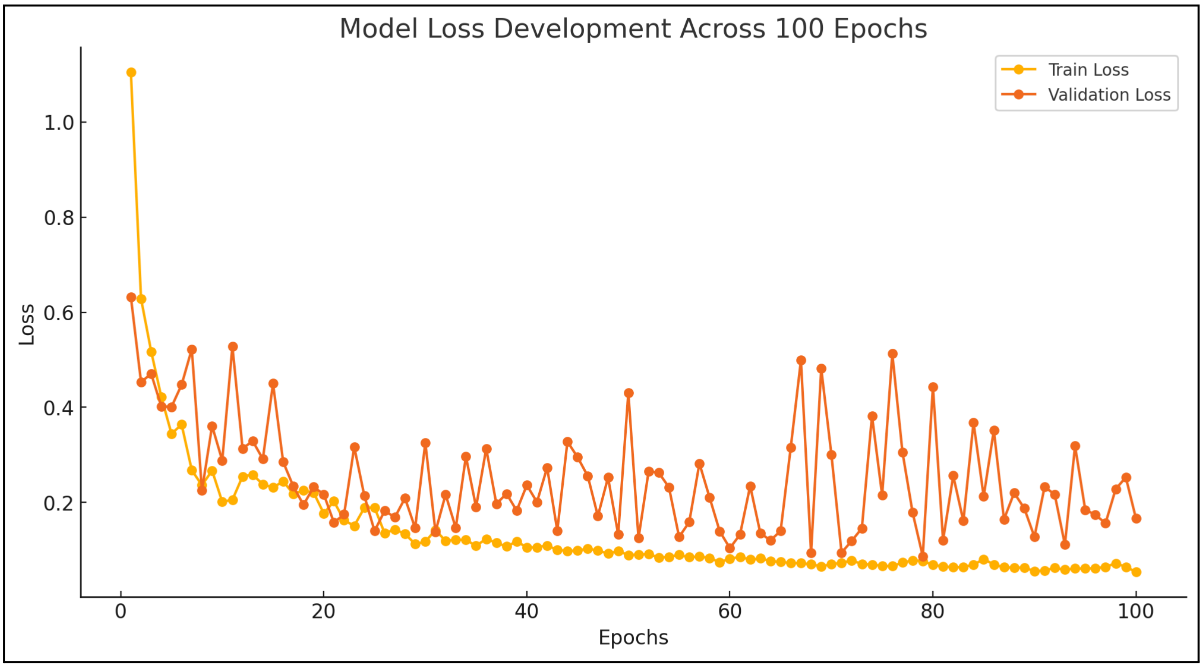

Figure S4. Model Loss development across 100 epochs. The plot illustrates the progression of both training loss and validation loss over the course of 100 epochs during the model's training process. The training loss, represented by yellow dots, reflects the error rate of the training dataset, while the validation loss, depicted as orange dots, indicates the error rate of the validation dataset. Initially, both loss metrics decrease significantly, showcasing effective learning by the model.

After the training was completed, the model was validated using 140 images by comparing its raw output against each corresponding image (Fig. S5). This validation process involved not only assessing how accurately the model identified and predicted the ROI but also classifying the images into two categories: axol_limb (late-regenerating axolotl limbs) and axol_early_limb (early-regenerating axolotl limbs). Upon successful validation, the model was saved in the H5 format and stored in the Meandros model directory, enabling it to be loaded within the graphical user interface (Fig. S1).

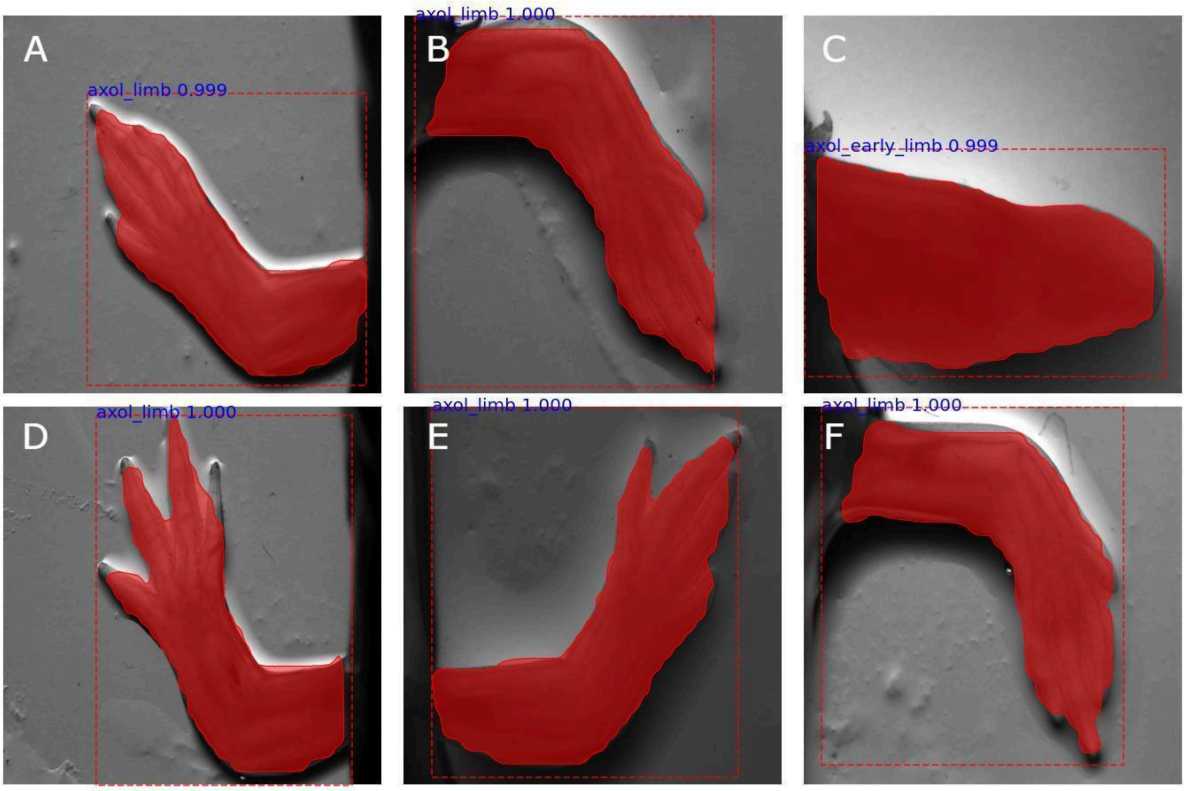

Figure S5. Examples of Meandros's performance when trained on the provided dataset. A-F) Meandros successfully identified the ROI (red) and classified the limb types (indicated by the blue legends). The process of loading a model into the cache of the computer involves several steps. Firstly, the user must ensure that the model is in the appropriate format, such as a .h5 file, that is compatible with the software. Once the model is selected, the software will then load it into memory. This process may take some time, depending on the size of the model and the computational resources of the computer. Once the model is loaded, it can be easily swapped with the pre-trained models or with other custom models in the cache. This allows for quick and efficient switching between models without the need to constantly reload them from disk. The loading of the model in memory also significantly reduces the response time when processing images for ROI detection. This is because the model does not need to be loaded from disk every time an image is processed, resulting in faster processing times.

2.2 Thresholding and Area exclusion

We followed standard thresholding to analyse the GFP-dependent fluorescence intensity in the microscopy images of the axolotl limbs during regeneration. The pixels with an intensity value greater than the threshold value were set to 1, while the pixels with a value less than the threshold value were set to 0. Typically, thresholding is enough to discriminate the foreground while filtering out weak signals that may be due to scattering artifacts or autofluorescence (Fig. S6*)*. In cases where the simple threshold is insufficient for this discrimination, Meandros features a tool called Exclude Regions, through which regions that are to be avoided in subsequent analyses, in particular regions affected by artifacts, can be manually selected.

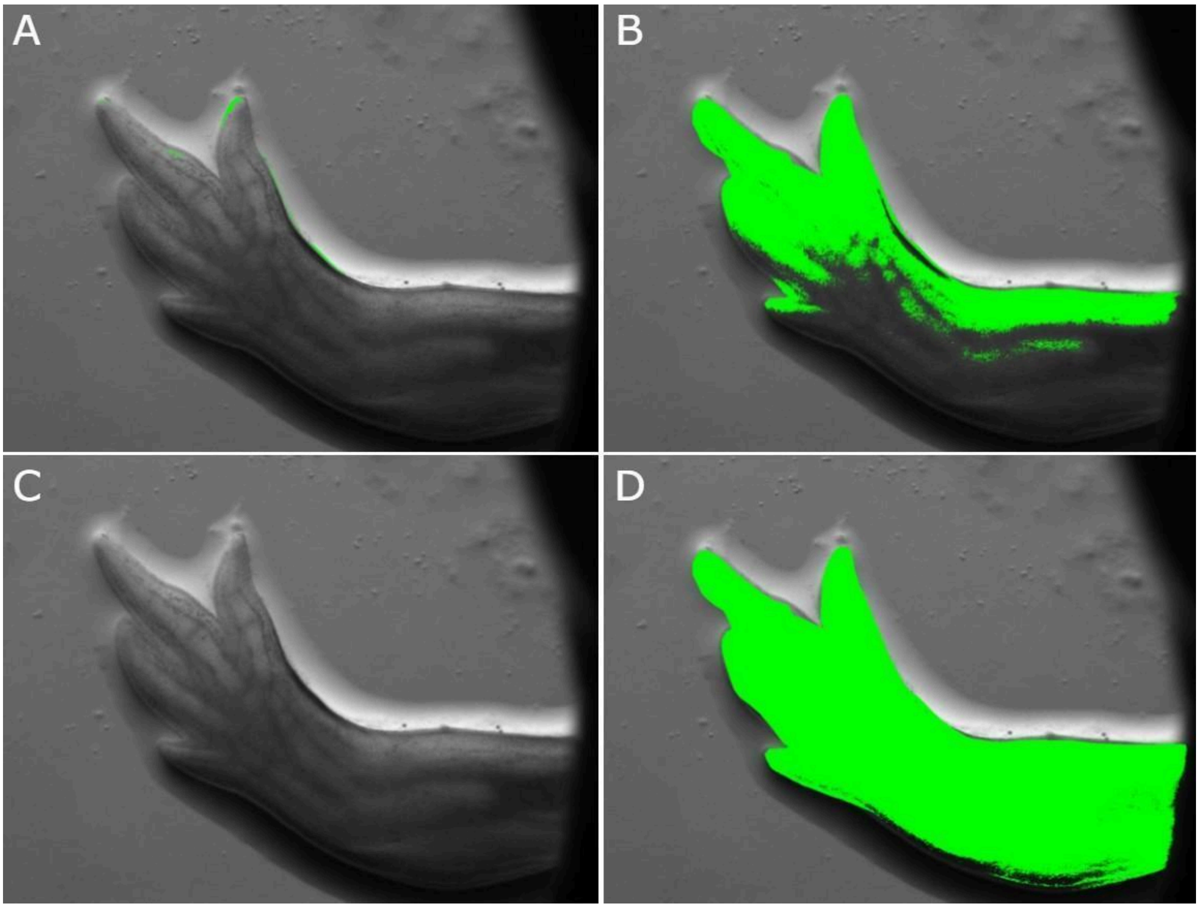

Figure S6. Thresholding. A) Brightfield channel of an axolotl's limb during regeneration. **B)** The image depicted in A) is processed by Meandros with a threshold value of 158 using the GFP channel. **C)** Same as B), but using a 95 threshold. **D)** Same as before but using a threshold of 26.

2.3 Axis detection

To quantify the density spatial profiles along the PD axis of the axolotl limbs, this spatial axis needs to be first specified. Meandros features a tool for defining the axis through which the intensity profile is to be extracted. The Axis detection module (Fig. S7) in the Meandros software utilizes the region obtained by the model selected by the user. The algorithm used in this module is based on the idea of approximating the axis and providing a first approach that requires minimal user intervention to correct the output of the module.

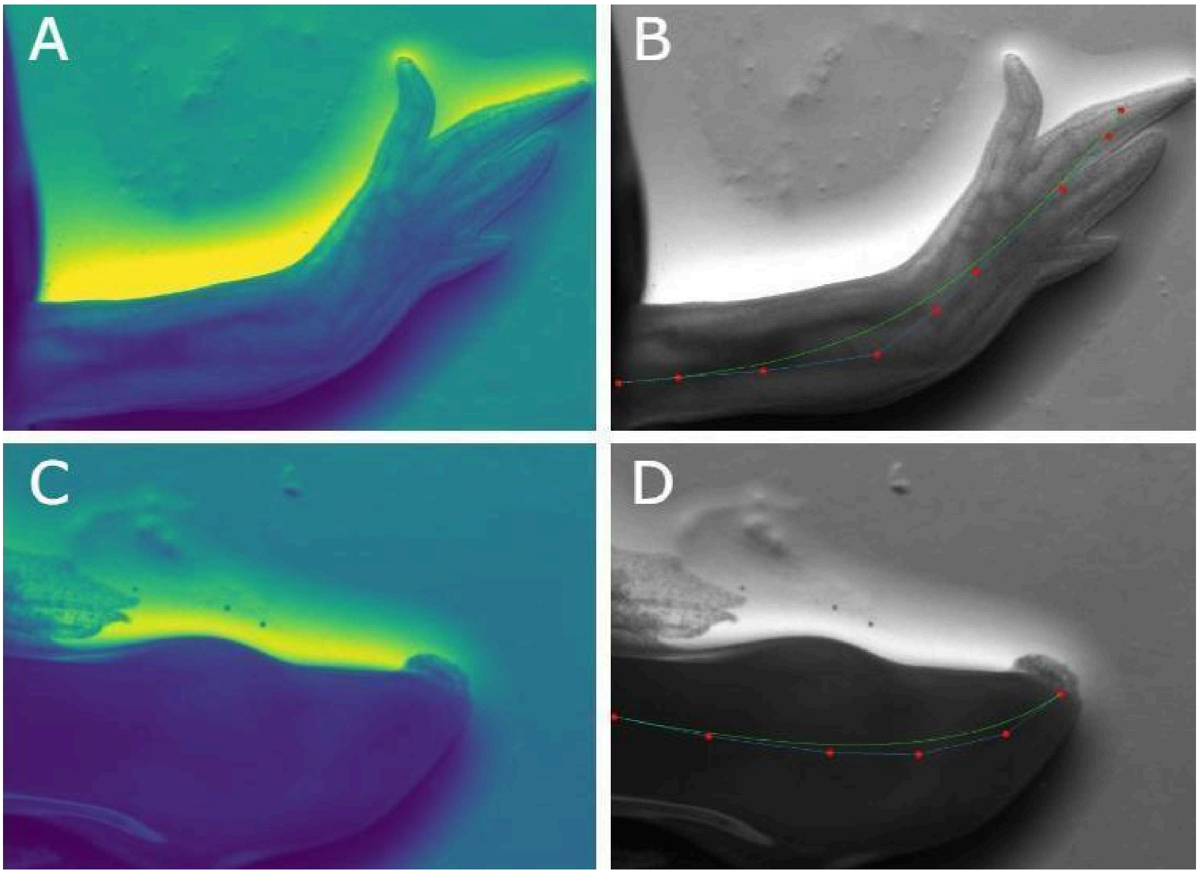

Figure S7. Proximal-distal (PD) axis detection. **A-B)** An axolotl limb (A) and the corresponding axis calculated using the Meandros software, supplemented by minor manual corrections (B). The green line corresponds to the PD axis, represented by a Bézier curve that smooths the trajectory defined by red points, which mark key anatomical landmarks. The blue segments indicate the interpolated sections used to achieve an optimized axis representation. **C-D**) Same as A-B) for an axolotl limb in the early stages of regeneration

First, the algorithm determines whether the contour's maximum span is along the X or Y axis of the image by identifying the farthest points in each direction. Once the dominant orientation is established, the contour points are divided into two halves, which are analysed separately to facilitate axis approximation. Once the dominant axis is established, the contour points are divided into two halves, which are analysed separately to facilitate axis approximation (Fig. S8*)*. The contour is segmented into discrete bins along the dominant-axis, assigning bin labels to categorize the points for further analysis. Within each bin, the extreme X values are identified and marked as reference points to aid in the generation of the approximated axis. The final axis representation is constructed by averaging corresponding points from both contour halves, resulting in a smoothed trajectory that accurately follows the limb's shape. Additional computational steps ensure that the contour points are processed sequentially, identifying the closest neighbouring points to maintain continuity.

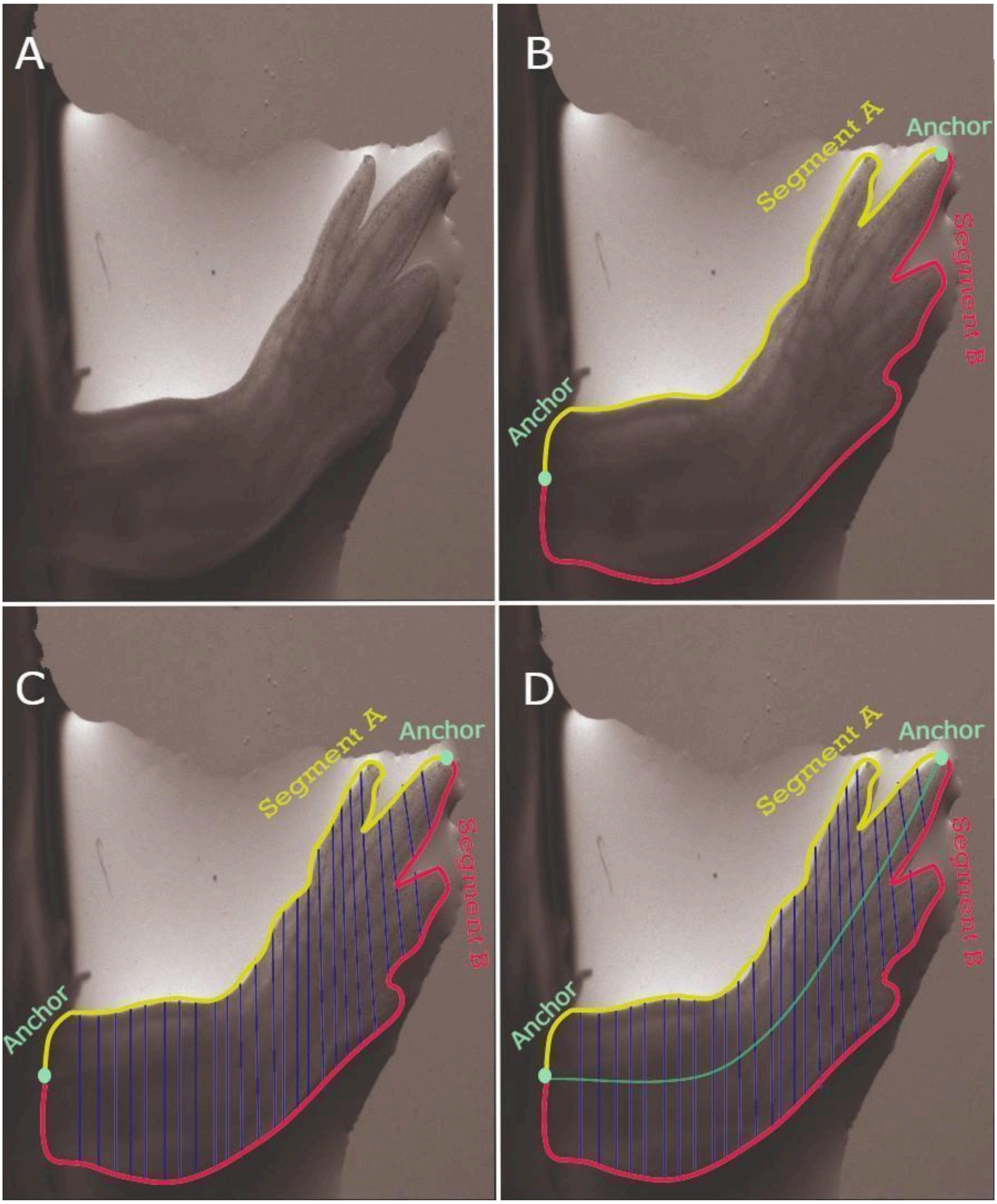

Figure S8. Proximal-distal (PD) Axis detection algorithm. A) Raw image of a regenerating axolotl limb. The ROI obtained with Meandros serves as the input for the algorithm of axis detection. **B)** By defining the extreme anchor points, the segment A (yellow) and B (red) are calculated. **C)** A binarization strategy divides the contour into multiple and equal segments covering the entire limb. **D)** The PD axis (green) is obtained through the whole ROI, between both anchor points (green dots).

After defining the main segments, the function divides the points within each segment into smaller groups of 25 units. It assigns a unique bin identifier to each group by comparing the values to the defined ranges. For each smaller segment, it determines whether a point belongs to that range, effectively binarizing the data based on the minimum and maximum values of each group along the y-axis or the x-axis depending on which axis was used to generate the segments before. This process

is repeated for all points in both Segment A and Segment B. With the binarization in place, the last step of the algorithm takes the middle point between the minimum value of each binarization segment of each segment A and B, and uses those points joined by a Bezier curve to estimate the axis that goes through the whole contour.

This algorithm is an example of a contour-based approach for approximating an axis within an image, it can be compared with other approaches like Hough Transform [7], Radon Transform [8] and others, which are also used in the literature for similar purposes.

2.4 Amputation Plane

Meandros enables users to define the amputation plane in the axolotl limb. Users can annotate a few key points along the amputation plane, which Meandros automatically connects using a Bézier curve. If needed, users can further refine the curve by moving, removing, or adding points to improve accuracy.

2.5 Landmarks

Meandros includes a module that allows users to create precise landmark annotations, with the flexibility to add, modify, or delete landmarks as needed to ensure precise alignment. These landmarks are later utilized in the Profiling module. In this study, we marked the amputation plane, elbow, and wrist as key landmarks.

2.6 Profiling module

The Profiling module in Meandros employs an algorithm for automated cell quantification based on fluorescence intensity along the proximodistal (PD) axis. The algorithm calculates the number of pixels with intensity values above the threshold and normalises this frequency (f) by the maximum fluorescence intensity along the anatomical axis (f/f_{max}). This ratio represents the relative fluorescence intensity for each position along the PD axis. The module also generates a graphical output showing the distribution of normalised density along the PD axis, with shaded regions indicating the standard deviation and a coloured centre line representing the mean (See Fig. 2 and 3 of the main article). Additionally, it displays the standard deviation of PD positions corresponding to anatomical landmarks, such as the elbow (*E*) and wrist (*W*), providing insights into the variability of fluorescence intensity along the limb.

3. Reaction-Diffusion-Advection theory of proximalisation in the axolotl regenerating limb

3.1 Proximalisation as an advective process

In this study, we modelled the spatiotemporal distribution of cells in the regenerating axolotl limb. Since, in general, cell diameters are approximately 10^{-3} times the size of the limb or smaller, we decided to adopt a continuous formalism and represent the dynamics of the density of cells within the limb tissue during regeneration (ρ) as a reaction-diffusion-advection process:

$$\frac{d\rho}{dt} = r\rho + D\frac{\partial^2\rho}{\partial x^2} - \frac{\partial(v_a\rho)}{\partial x} \quad \text{(Supp. Eq. 1)}$$

Where x represents the position within the PD axis of the regenerating limb. As described in the main text, the first term encodes a reaction term and includes the net average proliferation rate of the limb tissues r. This parameter can be associated with the net average cell cycle length as follows:

$$r = \frac{\ln(2)}{T_C}$$

The second term models cell diffusion within the limb and involves the cell diffusion coefficient D. Finally, the last term models the fact that the cells move within a tissue that expands, which we acknowledge with an advective velocity v_a.

$$\frac{d\rho}{dt} = r\rho + D\frac{\partial^2\rho}{\partial x^2} - \rho\frac{\partial v_a}{\partial x} - v_a\frac{\partial\rho}{\partial x} \quad \text{(Supp. Eq. 2)}$$

Because we know that the regenerating limb grows exponentially:

$$v_a = ax \quad \text{(Supp. Eq. 3)}$$

$$\frac{d\rho}{dt} = r\rho + D\frac{\partial^2\rho}{\partial x^2} - a\rho - ax\frac{\partial\rho}{\partial x} \quad \text{(Supp. Eq. 4)}$$

$$\frac{d\rho}{dt} = (r - a)\rho + D\frac{\partial^2\rho}{\partial x^2} - ax\frac{\partial\rho}{\partial x} \quad \text{(Supp. Eq. 5)}$$

We assume that the electroporated cells are subject to the same processes we described in Supp. Eqs. 1 to 5, with an additional advective velocity v_p oriented towards proximal regions that we denominate proximalisation velocity:

$$\frac{d\rho}{dt} = r\rho + D\frac{\partial^2\rho}{\partial x^2} - \frac{\partial(v_a\rho)}{\partial x} + \frac{\partial(v_p\rho)}{\partial x} \quad \text{(Supp. Eq. 6)}$$

$$\frac{d\rho}{dt} = r\rho + D\frac{\partial^2\rho}{\partial x^2} - \rho\frac{\partial v_a}{\partial x} - v_a\frac{\partial\rho}{\partial x} + \rho\frac{\partial v_p}{\partial x} + v_p\frac{\partial\rho}{\partial x} \quad \text{(Supp. Eq. 7)}$$

$$\frac{d\rho}{dt} = r\rho + D\frac{\partial^2\rho}{\partial x^2} - \rho a - ax\frac{\partial\rho}{\partial x} + \rho\frac{\partial v_p}{\partial x} + v_p\frac{\partial\rho}{\partial x} \quad \text{(Supp. Eq. 8)}$$

Assuming that the proximalisation velocity is constant along the PD axis, $\frac{\partial v_p}{\partial x} = 0$

$$\frac{d\rho}{dt} = r\rho + D\frac{\partial^2\rho}{\partial x^2} - \rho a - ax\frac{\partial\rho}{\partial x} + v_p\frac{\partial\rho}{\partial x} \quad \text{(Supp. Eq. 9)}$$

$$\frac{d\rho}{dt} = (r - a)\rho + D\frac{\partial^2\rho}{\partial x^2} - ax\frac{\partial\rho}{\partial x} + v_p\frac{\partial\rho}{\partial x} \quad \text{(Supp. Eq. 10)}$$

$$\frac{d\rho}{dt} = (r - a)\rho + D\frac{\partial^2\rho}{\partial x^2} + \left(v_p - ax\right)\frac{\partial\rho}{\partial x} \qquad \text{(Supp. Eq. 11)}$$

which corresponds to the Eq. 7 of the main article (See section 2.3)

3.2 Proximalisation as a force

Using the Smoluchowski diffusion theory (see, for instance, https://en.wikipedia.org/wiki/Convection-diffusion_equation), we can express the proximalisation velocity in terms of a proximalisation force, pointing towards proximal regions, that is, with the same sign than the v_p:

$$\frac{d\rho}{dt} = (r - a)\rho + D\frac{\partial^2\rho}{\partial x^2} + \left(\mu F_p - ax\right)\frac{\partial\rho}{\partial x} \qquad \text{(Supp. Eq. 12)}$$

Where μ is the mobility of the electroporated cells when subjected to the proximalisation force. This proximalisation force is illustrated as the slope of the proximalisation potential lines depicted in Figure 6.

3.3 Proximalisation as a potential

If we assume that the proximalisation force is conservative, from classical mechanics, we can write the force as follows:

$$F =- \nabla U(x) =- \frac{\partial U}{\partial x}(x) \qquad \text{(Supp. Eq. 13)}$$

$$dU =- Fdx$$

$$\int_{U(x=0)\equiv U_0}^{U(x)} dU =- \int_0^x Fdx$$

Where $x = 0$ would correspond to the shoulder of the axolotl. Since $F =- F_p$

$$U_p(x) = U_p^0 + F_p x \qquad \text{(Supp. Eq. 14)}$$

Where U_p is the proximalisation potential that is a linear function of the PD axis position x. This proximalisation potential is represented in Figure 6.

3.4 Proximalisation velocity as a chemotaxis-driven process

By using classical models of chemotaxis [9], we can express Supp. Eq. 10 in terms of a chemotaxis process. If the advective flux emerges from chemotaxis, the last term of Supp. Eq. 10 $v_p\frac{\partial\rho}{\partial x}$ can be re-written as $-\chi\frac{\partial}{\partial x}\left(\rho\frac{\partial c}{\partial x}\right)$, where c is the concentration of the chemotactic species and χ is the chemotaxis strength. The only possibility for this correspondence to occur is to assume that c linearly decays with the PD position x:

$$c(x) = c_0 - \theta x \qquad \text{(Supp. Eq. 15)}$$

Meaning that

$$\frac{\partial c}{\partial x} = - \theta$$

As a consequence:

$$v_p = \chi\theta = \chi\left(- \frac{\partial c}{\partial x}\right) \qquad \text{(Supp. Eq. 16)}$$

That is, in the chemotaxis version of the model, the proximalisation velocity is the product of the chemotaxis strength and the negative gradient of the chemotactic species concentration gradient

This would mean that the proximalisation force can written in terms of the chemotaxis process as:

$$F_p = \frac{\chi\theta}{\mu} \qquad \text{(Supp. Eq. 17)}$$

And, finally, the proximalisation potential could be written as:

$$U_p(x) = U_p^0 + \left(\frac{\chi\theta}{\mu}\right)x \qquad \text{(Supp. Eq. 18)}$$

4. References

[1] Córdoba, R., Arce, H., and Chara, O. (2025). Meandros (1.0.0). Zenodo. https://doi.org/10.5281/zenodo.15036317

[2] He, K., Gkioxari, G., Dollár, P. and Girshick, R. (2017) Mask R-CNN. IEEE International Conference on Computer Vision (ICCV), 2980–2988.

[3] He, K., Xiangyu, Z., Shaoqing, R. and Jian, S. (2016) Deep residual learning for image recognition. IEEE Conference on Computer Vision and Pattern Recognition, 770–778.

[4] Abdulla W. 2017. "Mask R-CNN for object detection and instance segmentation on Keras and TensorFlow". GitHub repository. https://github.com/matterport/Mask_RCNN.

[5] Dutta, A. and Zisserman, A. (2019) The VIA Annotation Software for Images, Audio and Video. 27th ACM International Conference on Multimedia (MM '19), Nice, France, 4 pages.

[6] Lin, T. Y., Maire, M., Belongie, S., Bourdev, L., Girshick, R., Hays, J., Perona, P., Ramanan, D., Lawrence, C. and Dollár, P. (2015) Microsoft COCO: Common Objects in Context.

[7] Duda, R. O. and Hart, P. E. (1972) Use of the Hough transformation to detect lines and curves in pictures. Commun. ACM 15(1), 11–15.

[8] Radon, J. (1986) On the determination of functions from their integral values along certain manifolds. IEEE Trans. Med. Imaging.

[9] Murray, J. D. (2002) Mathematical Biology (3rd ed.). Springer.
